# Heme acts through the Bach1b/Nrf2a-MafK pathway to regulate exocrine peptidase precursor genes in porphyric zebrafish

**DOI:** 10.1242/dmm.014951

**Published:** 2014-03-20

**Authors:** Shuqing Zhang, Minrui Xu, Jian Huang, Lili Tang, Yanqing Zhang, Jingyao Wu, Shuo Lin, Han Wang

**Affiliations:** 1 Center for Circadian Clocks, Soochow University, Suzhou 215123, Jiangsu, China.; 2 School of Biology & Basic Medical Sciences, Medical College, Soochow University, Suzhou 215123, Jiangsu, China.; 3 Department of Molecular, Cell and Developmental Biology, University of California, Los Angeles, CA 90095-1606, USA.

**Keywords:** Bach1b, Nrf2a, MafK, Heme, Porphyria, Zymogens, Zebrafish

## Abstract

Using a zebrafish model of hepatoerythropoietic porphyria (HEP), we identify a previously unknown mechanism underlying heme-mediated regulation of exocrine zymogens. Zebrafish *bach1b*, *nrf2a* and *mafK* are all expressed in the zebrafish exocrine pancreas. Overexpression of *bach1b* or knockdown of *nrf2a* result in the downregulation of the expression of the exocrine zymogens, whereas overexpression of *nrf2a* or knockdown of *bach1b* cause their upregulation. *In vitro* luciferase assays demonstrate that heme activates the zymogens in a dosage-dependent manner and that the zymogen promoter activities require the integral Maf recognition element (MARE) motif. The Bach1b-MafK heterodimer represses the zymogen promoters, whereas the Nrf2a-MafK heterodimer activates them. Furthermore, chromatin immunoprecipitation (ChIP) assays show that MafK binds to the MARE sites in the 5′ regulatory regions of the zymogens. Taken together, these data indicate that heme stimulates the exchange of Bach1b for Nrf2a at MafK-occupied MARE sites and that, particularly in heme-deficient porphyria, the repressive Bach1b-MafK heterodimer dominates, which can be exchanged for the activating Nrf2a-MafK heterodimer upon treatment with hemin. These results provide novel insights into the regulation of exocrine function, as well as the pathogenesis of porphyria, and should be useful for designing new therapies for both types of disease.

## INTRODUCTION

As the prosthetic moiety for numerous proteins and enzymes – such as hemoglobin, catalases and cytochrome – heme is essential for nearly all forms of life through oxygen transport, respiration, detoxification and other important processes (Padmanaban et al., 1989; Igarashi and Sun, 2006; Mense and Zhang, 2006; Balwani and Desnick, 2012). A cascade of eight highly conserved enzymatic reactions controls heme biosynthesis (Kappas et al., 1995; Ryter and Tyrrell, 2000; Balwani and Desnick, 2012), and a group of rare and clinically complex diseases, known as porphyria, result directly from various genetic mutations that cause defective enzymes in the heme biosynthetic pathway (Kappas et al., 1995; Bonkovsky, 2005; Solinas and Vajda, 2008; Cappellini et al., 2010; Puy et al., 2010; Siegesmund et al., 2010; Balwani and Desnick, 2012). We have previously established a heme-deficient zebrafish that is homozygous for a mutation in *uroporphyrinogen decarboxylase* (*urod*; also known as *yquem*) as a model for studying human hepatoerythropoietic porphyria (HEP) (Online Mendelian Inheritance in Man number 176100) and heme deficiency pathogenesis (Wang et al., 1998). Our microarray and *in situ* hybridization analyses of this zebrafish HEP model revealed downregulation of six peptidase precursor genes – including *carboxypeptidase A*5 (*cpa5*), *chymotrypsinogen 1 like* (*ctr1l*), *chymotrypsinogen B1* (*ctrb1*), *elastase 2 like* (*ela2l*), *trypsin precursor* (*try*) and *trypsin like* (*tryl*) – specifically in the exocrine pancreas of the HEP zebrafish (*yquem/urod*, *−/−*) (Wang et al., 2007). Of these six zymogens, *cpa5* (previously called *cpa*) belongs to the MEROPS peptidase family M14, whereas the other five peptidases contain a trypsin-like serine protease (tryp_SPc) domain and are members of the serine peptidase chymotrypsin family S1 (chymotrypsin A, clan PA) (http://merops.sanger.ac.uk/) (Wang et al., 2007). These serine peptidases function extracellularly to aid food digestion (Hedstrom, 2002; Wang et al., 2007). However, the molecular mechanism underlying how these exocrine zymogens regulate heme remains poorly understood (Wang et al., 2007).

**Fig. 1.**
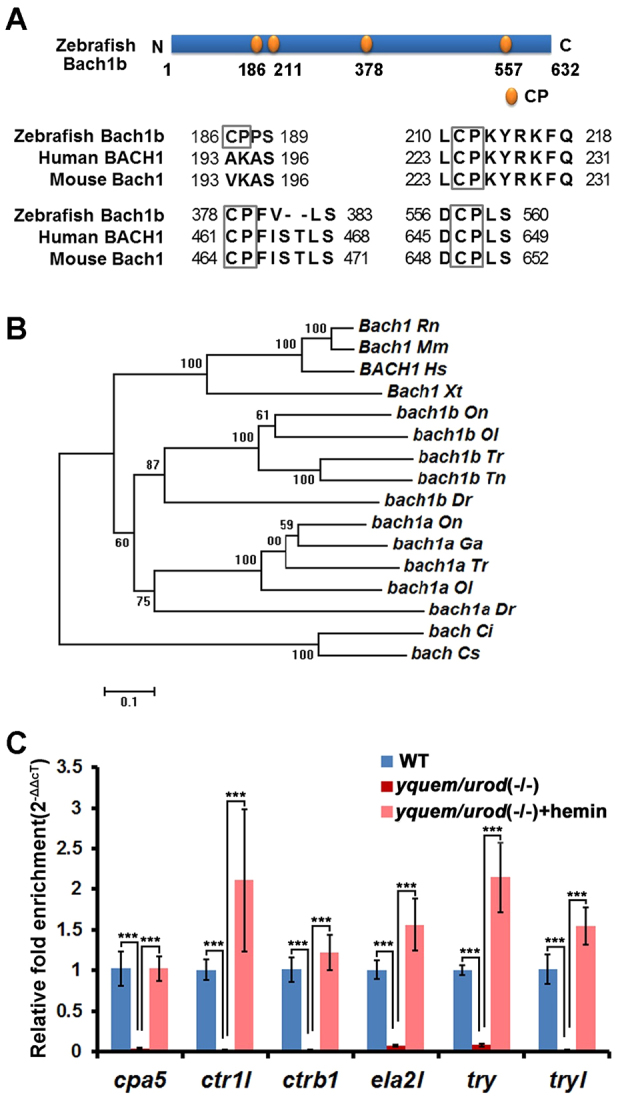
**Zebrafish *bach1b* is a co-ortholog of mammalian *Bach1*, and heme regulates exocrine peptidase precursor genes.** (A) Zebrafish Bach1b contains four CP motifs, three of which are also found in human and mouse. (B) Phylogenetic analysis of Bach1 proteins shows that zebrafish contain two *bach1* genes, *bach1a* and *bach1b*, which are co-orthologs of mammalian *Bach1*. The tree was constructed using the neighbor-joining method with MEGA5 (Tamura et al., 2011). The numbers indicate the percentage bootstrap support. *Dr*, *Danio rerio; Tr*, *Takifugu rubripes; Tn*, *Tetraodon nigroviridis; Ol*, *Oryzias latipes; Ga*, *Gasterosteus aculeatus; On*, *Oreochromis niloticus; Xt*, *Xenopus tropicalis; Ci*, *Ciona intestinalis; Cs*, *Ciona savignyi; Rn*, *Rattus norvegicus; Mm*, *Mus musculus; Hs*, *Homo sapiens. Ciona intestinalis* and *Ciona savignyi* Bach proteins were used as an outgroup. The Ensembl gene IDs of these genes are listed in supplementary material Table S2. (C) Downregulation of six peptidase precursor genes in zebrafish *yquem/urod* (−/−) and their rescue upon treatment with hemin. The concentration of hemin used was 100 μM. The expression levels of six peptidase precursor genes in larvae at 84 hpf were determined by using qRT-PCR. Student’s *t*-tests were conducted. *P<0.05, **P<0.01, ***P<0.001.

Heme also serves as a signaling molecule and modulates a number of molecular and cellular processes (Sassa and Nagai, 1996; Sun et al., 2004; Mense and Zhang, 2006). Several studies have shown that heme regulates gene expression by mediating the dynamic exchange of BTB and cap‘n’collar homolog 1 (Bach1) and nuclear factor erythroid 2 p45-related factor 2 (Nrf2) in the small musculoaponeurotic fibrosarcoma (Maf) oncogene transcription factor network (Igarashi et al., 1998; Ogawa et al., 2001; Kitamuro et al., 2003; Brand et al., 2004; Sun et al., 2004; Marro et al., 2010). MafK is a member of the small Maf protein family that binds to the Maf Recognition Element (MARE) motif [TGCTGA(C/G)TCAGCA] through its DNA-binding basic leucine zipper (bZIP) domain (Toki et al., 2005; Igarashi and Sun, 2006; Blank, 2008). However, because MafK lacks a transactivation domain, it regulates transcription through interaction with the cap‘n’collar (CNC) bZIP proteins, including Nrf2 and Bach1 (Toki et al., 2005; Igarashi and Sun, 2006; Blank, 2008). The Nrf2-MafK heterodimer serves as an activator (Pratt et al., 2002; Kobayashi and Yamamoto, 2006), whereas the Bach1-MafK heterodimer functions as a repressor because Bach1 harbors a transcription-repressing BTB/POZ domain (Toki et al., 2005; Igarashi and Sun, 2006; Blank, 2008). Moreover, heme binds to Bach1 through its cysteine-proline (CP) dipeptide-containing heme regulatory motifs (HRMs) (Fig. 1A) (Kitamuro et al., 2003). Heme-binding dissociates Bach1 from MafK, thereby abolishing repression of its downstream genes (Ogawa et al., 2001; Kitamuro et al., 2003; Sun et al., 2004; Suzuki et al., 2004; Marro et al., 2010).

**TRANSLATIONAL IMPACT**

**Clinical issue**

The iron-containing heme group is required for the biological function of numerous proteins and enzymes and is essential for nearly all forms of life. A cascade of eight highly conserved enzymatic reactions controls heme biosynthesis. Various mutations in the genes encoding the proteins required for these reactions cause a group of rare and clinically complex metabolic diseases that are known as porphyria. Human hepatoerythropoietic porphyria (HEP) is a rare form of porphyria that is characterized by the severe blistering of skin that is exposed to sunlight. A previous transcriptome analysis of a zebrafish model for HEP revealed an unexpected aspect of porphyria – the underproduction of a group of pancreatic zymogens, inactive forms of digestive enzymes that are secreted by the pancreas and subsequently activated in the intestine. How heme regulates the transcription of these pancreatic zymogens is unknown.

**Results**

In this study, the authors use the zebrafish model for HEP and a combination of genetic, embryological, *in vitro* cell transfection and biochemical approaches to investigate the regulation of pancreatic zymogen production by heme. They show that heme determines pancreatic zymogen production levels by mediating the dynamic exchange of the bZIP transcription factors Bach1b and Nrf2a at Maf recognition element (MARE) sites that are occupied by MafK, another bZIP transcription factor. Specifically, in conditions of heme-deficiency such as porphyria, the Bach1b-MafK heterodimer occupies the MARE sites of the genes encoding pancreatic zymogens and represses their expression. Notably, the authors show that treatment with hemin (a reddish-brown chloride of heme) can dissociate Bach1b from MafK, which allows Nrf2a to enter the MafK-occupied MARE sites. The Nrf2a-MafK heterodimer, which is a transcriptional activator, then restores zymogen production.

**Implications and future directions**

These findings shed light on the frequently reported acute episodic abdominal pains with co-occurrences of nausea and vomiting in some porphyria individuals, and on the regulation of exocrine function. Specifically, because the findings reported here also show that the roles of the Bach1b, Nrf2a and MafK in heme-mediated regulation of exocrine zymogens appear to be highly conserved between zebrafish and human, the acute episodic abdominal pains in these individuals probably stem from decreased production of pancreatic zymogens. Moreover, these findings provide an explanation for why treatment with hemin can cure these symptoms in individuals with porphyria and identify *BACH1* expression in the exocrine pancreas as a potential new therapeutic target.

Thus, we hypothesize that under heme-deficient conditions, such as in porphyria, the Bach1-MafK heterodimer binds to the MARE sites of exocrine pancreatic zymogens and represses their transcription. Conversely, at normal or higher levels of heme (induced by hemin treatment, for instance), heme binds to Bach1 and triggers the dissociation of Bach1 from MafK, allowing Nrf2 to enter the MafK-occupied MARE sites in the regulatory regions of zymogens and then activate them.

To test this hypothesis, zebrafish *bach1b* (a co-ortholog of mammalian *Bach1*) (Fig. 1B) was isolated, its expression, along with the expression of zebrafish *nrf2a* (a co-ortholog of mammalian *Nrf2*) (Timme-Laragy et al., 2012) and *mafK* (Takagi et al., 2004), in the zebrafish exocrine pancreas was determined; the effect of both knockdown and overexpression of *bach1b* or *nrf2a* on the expression of *cpa5*, *ctr1l*, *ctrb1*, *ela2l*, *try* and *tryl* was examined, and *in vitro* cell transfection and chromatin immunoprecipitation (ChIP) assays were conducted to elucidate how heme regulates these zymogens. Our studies reveal novel aspects of heme deficiency pathogenesis and should offer new ways to diagnose and treat individuals that have porphyria.

## RESULTS

### Heme regulation of exocrine peptidase precursor genes

Our previous microarray and whole-mount *in situ* hybridization analyses of the zebrafish *yquem/urod* (*−/−*) HEP model shows that six peptidase precursor genes – *cpa5*, *ctr1l*, *ctrb1*, *ela2l*, *try* and *tryl* – are significantly downregulated in this zebrafish model (Wang et al., 2007). Quantitative real-time (qRT)-PCR analysis corroborated these previous findings (Fig. 1C). Remarkably, treatment with hemin (a reddish-brown chloride of heme) significantly restored the expression of these six peptidase precursor genes in the zebrafish *yquem/urod* (*−/−*) (Fig. 1C) (Wang et al., 2007). These results indicate that heme regulates transcription of these six exocrine zymogens in zebrafish. Here, we aimed to elucidate its underlying molecular mechanism.

### Expression of *bach1b*, *nrf2a* and *mafK* in the exocrine pancreas

**Fig. 2.**
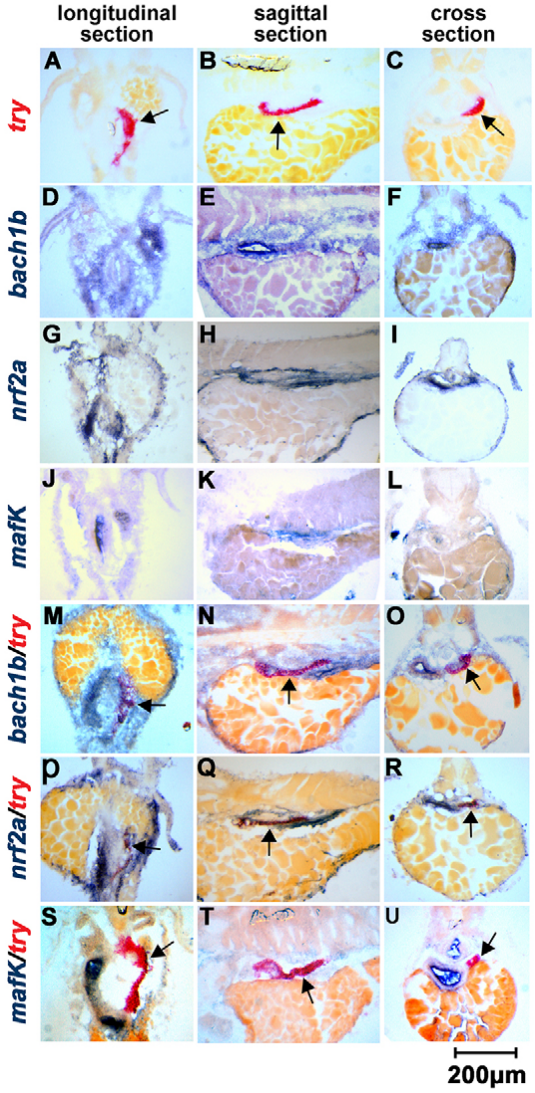
**Expression of *bach1b*, *nrf2a* and *mafK* in the exocrine pancreas as shown by using double *in situ* hybridization.** The fluorescein-labeled probe indicated the expression areas (red) of *try*, an exocrine pancreas marker (A,B,C). Digoxigenin-labeled probes indicated the expression areas (blue) of *bach1b* (D,E,F), *nrf2a* (G,H,I) and *mafK* (J,K,L). The colocalization of *try* with *bach1b* (M,N,O), *nrf2a* (P,Q,R), or *mafK* (S,T,U) in the exocrine pancreas was shown by using double *in situ* hybridization. Longitudinal sections are shown in A,D,G,J,M,P,S; sagittal sections are shown in B,E,H,K,N,Q,T; and cross-sections are shown in C,F,I,L,O,R,U. All larvae examined were at 84 hpf. Arrows indicate the exocrine pancreas.

Because we hypothesized that Bach1, Nrf2 and MafK are involved in heme-mediated regulation of these exocrine peptidase precursor genes, we first examined whether these three genes were expressed in the exocrine pancreas. We have previously examined two genes that were initially called zebrafish *bach1* and *nrf2*. Owing to the teleost genome duplication (Amores et al., 2011), zebrafish possess more copies of certain genes (Postlethwait et al., 2004; Wang, 2008). Through interrogation of fish genome sequences and phylogenetic analysis, we have recently found that zebrafish have two *bach1* genes – *bach1a* and *bach1b* – which are co-orthologs of mammalian *Bach1* (Fig. 1B); Timme-Laragy et al. have also reported that zebrafish have two *nrf2* genes – *nrf2a* and *nrf2b* – which are co-orthologs of mammalian *Nrf2* (Timme-Laragy et al., 2012). Therefore, the two genes that we have examined (here and previously) are actually zebrafish *bach1b* and *nrf2a*. Double *in situ* hybridization analyses showed that *bach1b*, *nrf2a* and *mafK* are all expressed in the exocrine pancreas, as evidenced by colocalization of the transcription of the three genes in the exocrine pancreas with the exocrine pancreas maker gene *try* (Wang et al., 2007) (Fig. 2); however, they also are expressed in some other tissues in zebrafish larvae. Because these results suggest that *bach1b*, *nrf2a* and *mafK* play regulatory roles in the exocrine pancreas, we focused on these three genes in this study.

### Roles of Bach1b and Nrf2a in regulation of exocrine peptidase precursor genes

**Fig. 3.**
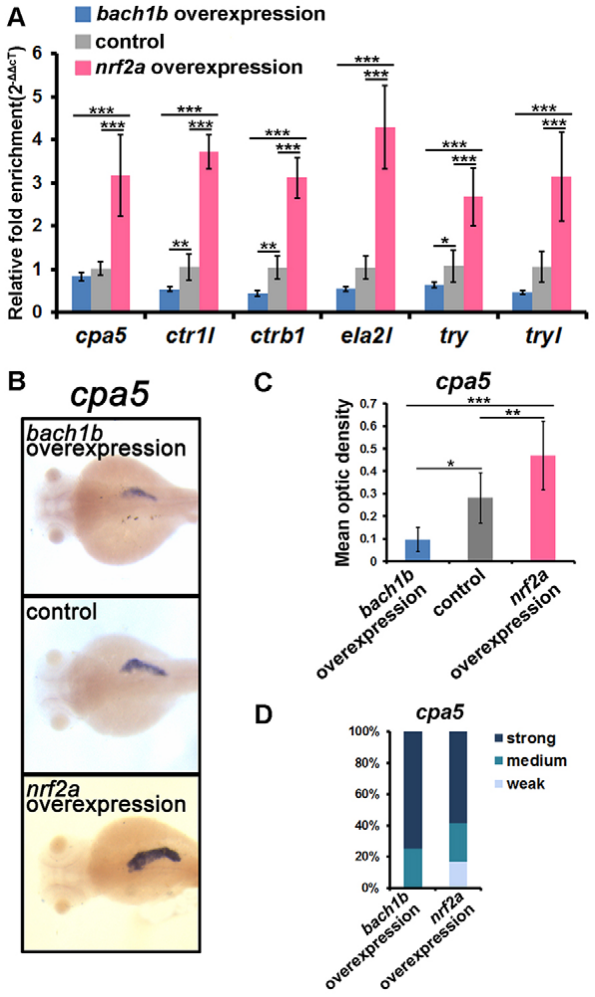
**Overexpression experiments with *bach1b* and *nrf2a* reveal their antagonism in the regulation of zymogen expression.** (A) qRT-PCR analysis showed that *bach1b* overexpression resulted in the downregulation of six peptidase precursor genes – *cpa5*, *ctr1l*, *ctrb1*, *ela2l*, *try* and *tryl* – whereas *nrf2a* overexpression resulted in their upregulation. Overexpression experiments were performed by microinjecting *bach1b* or *nrf2a* capped mRNAs into one-cell stage embryos. The expression levels of the six peptidase precursor genes in the microinjected and control larvae at 84 hpf were determined by qRT-PCR analysis. (B) Representative images of *in situ* hybridization staining show that downregulation of *cpa5* resulted from *bach1b* overexpression and that its upregulation resulted from *nrf2a* overexpression, both specifically in the exocrine pancreas. Dorsal view, anterior to the left. (C) Mean optic densities of *in situ* hybridization staining of a group of larvae (10–12 each) corresponding to Fig. 3B were quantified by using ImageJ. (D) *cpa5* morphant phenotypes. For individual larvae (84 hpf) of the *bach1b* overexpression group, the optic density values lower than the mean optic density value of its own group were marked as ‘strong’, and the optic density values higher than the mean optic density of the control group were marked as ‘weak’. For individual larvae of the *nrf2a* overexpression group, the optic density values higher than the mean optic density value of its own group were marked as ‘strong’, the optic density values lower than the mean optic density of the control group were marked as ‘weak’, and the values of optic density between ‘weak’ and ‘strong’ marked as ‘medium’. The statistical significance of difference between means was determined by one-way ANOVA and Tukey’s multiple comparison test (*n*=9) by using SPSS10.0.1. *P<0.05, **P<0.01, ***P<0.001.

**Fig. 4.**
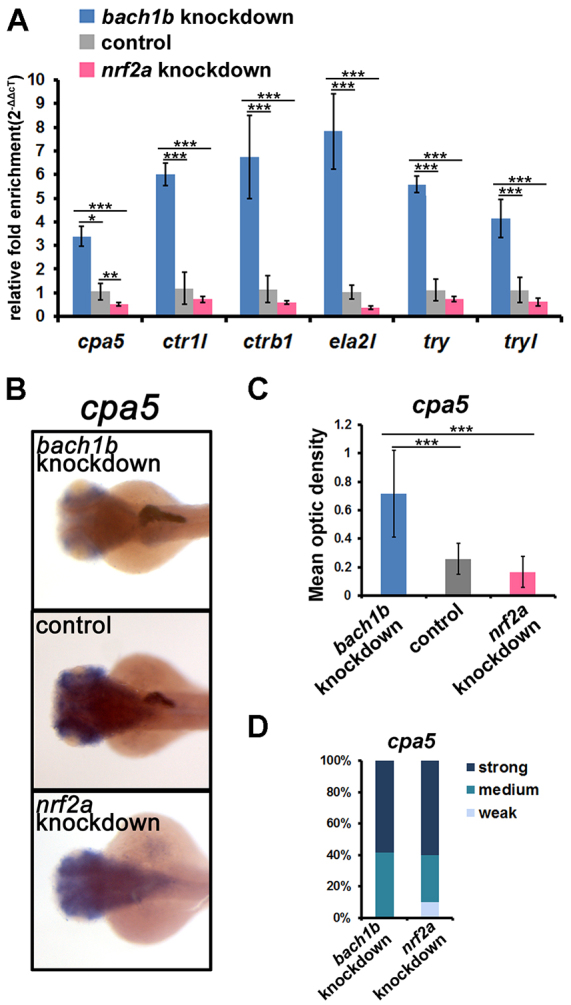
**Knockdown experiments with *bach1b* and *nrf2a* reveal their antagonism in the regulation of zymogen expression.** (A) qRT-PCR analysis showed that *bach1b* knockdown results in the upregulation of the six peptidase precursor genes that we examined, whereas *nrf2a* knockdown resulted in their downregulation. Knockdown experiments were performed by microinjecting MOs that targeted *bach1b* or *nrf2a* into one-cell stage embryos. For *bach1b* knockdown experiments, an ATG MO and SPL MO were used. Reverse transcription PCR showed that the SPL MO effectively altered *bach1b* intron 2 splicing (supplementary material Fig. S1A,B). The ATG MO also effectively knocked down *bach1b* (supplementary material Fig. S1C). The results using the SPL MO are shown here. For *nrf2a* knockdown experiments, we used an ATG MO, of which the efficacy of knockdown has been confirmed previously (Kobayashi et al., 2002; Kobayashi et al., 2009; Wang and Gallagher, 2013). The expression levels of the six peptidase precursor genes in the microinjected and control larvae at 84 hpf were determined by using qRT-PCR. (B) Representative images of *in situ* hybridization staining show that the upregulation of *cpa5* resulted from *bach1b* knockdown and that its downregulation resulted from *nrf2a* knockdown, both results occurred specifically in the exocrine pancreas. Dorsal view, anterior to the left. (C) Mean optic densities of *in situ* hybridization staining of a group of larvae (10–12 each) corresponding to Fig. 4C were quantified by using ImageJ. (D) *cpa5* morphant phenotypes. For individual larvae (84 hpf) of the *bach1b* knockdown overexpression group, the optic density values higher than the mean optic density value of its own group were marked as ‘strong’, and the optic density values lower than the mean optic density of the control group were marked as ‘weak’. For individual larvae of the *nrf2a* knockdown group, the optic density values lower than the mean optic density value of its own group were marked as ‘strong’, the optic density values higher than the mean optic density of the control group were marked as ‘weak’, and the values of optic density between ‘weak’ and ‘strong’ were marked as ‘medium’. The statistical significance of difference between means was determined by using one-way ANOVA and Tukey’s multiple comparison test (*n*=9) with SPSS10.0.1. *P<0.05, **P<0.01, ***P<0.001.

To elucidate the roles of Bach1b and Nrf2a in the regulation of exocrine zymogens, we conducted both overexpression and knockdown experiments. Capped mRNAs of *bach1b* and *nrf2a* (overexpression assays), or antisense oligonucleotide morpholinos (MOs) that were directed against *bach1b* and *nrf2a* (knockdown assays), were microinjected into one-cell stage embryos. Both qRT-PCR and *in situ* hybridization were then performed in order to determine the expression levels of the six peptidase precursor genes in the microinjected and control larvae. Both the *bach1b* ATG MO (targeting the start codon sequence) and the SPL (splicing) MO were used for *bach1b* knockdown. Reverse transcription PCR showed that the SPL MO effectively blocked the splicing of the second intron of *bach1b*, and DNA sequencing analysis revealed that part of the intron 2 sequence was transcribed and that a premature stop codon resulted in a short peptide of 88 amino acids (supplementary material Fig. S1A,B). We also showed that the ATG MO has a similar knockdown effect on *bach1b* to that of the SPL MO (supplementary material Fig. S1C). For knockdown of *nrf2a*, we used the *nrf2a* ATG MO, the efficacy of which has been confirmed previously (Kobayashi et al., 2002; Kobayashi et al., 2009; Wang and Gallagher, 2013). qRT-PCR showed that both *bach1b* overexpression and *nrf2a* knockdown result in downregulation of these six zymogens, whereas both *nrf2a* overexpression and *bach1b* knockdown caused their upregulation (Fig. 3A and Fig. 4A). Intriguingly, *in situ* hybridization showed that the downregulation or upregulation of the zymogen genes *cpa5*, *ctr1l*, *ctrb1*, *ela2l*, *try* and *tryl* occurred specifically in the exocrine pancreas (Fig. 3B–D; Fig. 4B–D; supplementary material Fig. S2 and Fig. S3A–C). These results suggest that Bach1b and Nrf2a function as a negative regulator and a positive regulator, respectively, in the regulation of these zymogens.

### Dosage-dependent activation of the promoters of exocrine peptidase precursor genes by heme

**Fig. 5.**
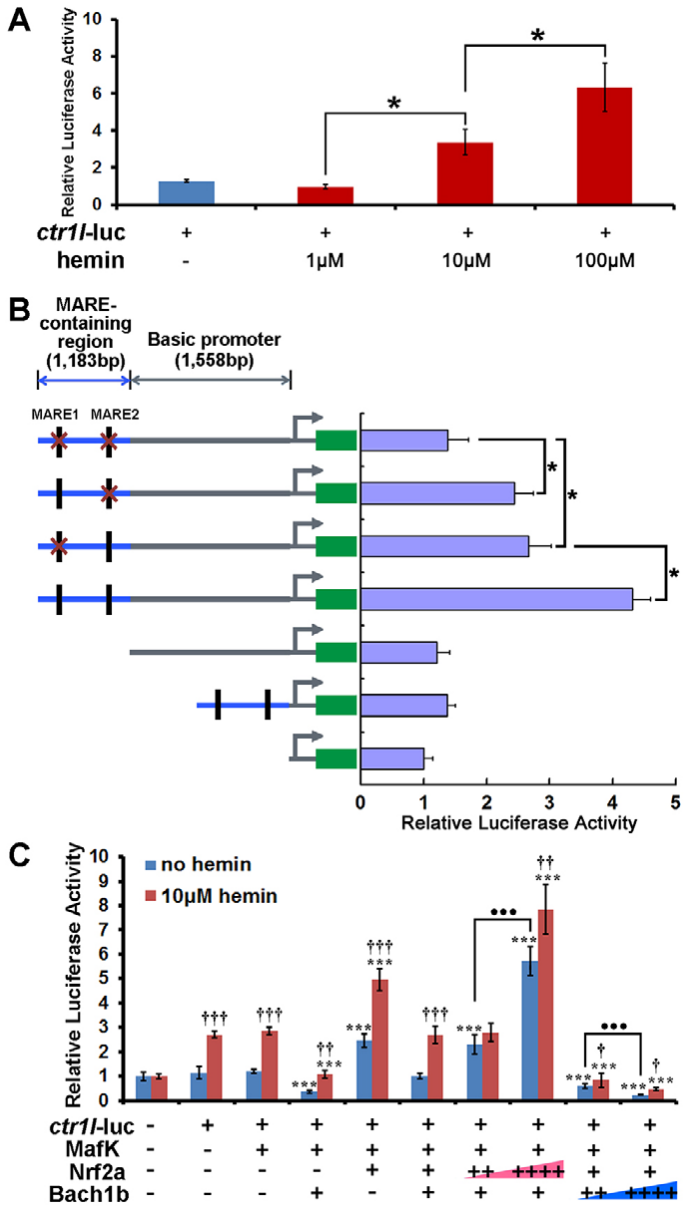
**MARE-dependent regulation of the *ctr1l* promoter activity by heme, Bach1b and Nrf2a.** (A) Activities of the *ctr1l* promoter increased with the addition of increasing concentrations of hemin. (B) The activity of the *ctr1l* promoter requires the presence of the MARE site – the *ctr1l* promoter with mutated MARE sites was not functional, and the *ctr1l* promoter fragment with two MARE motifs displayed stronger activity than that with only one MARE motif. Red crosses indicate mutated MARE sites. (C) Co-transfection of *mafK* and *nrf2a* enhances the activity of the *ctr1l* promoter, whereas co-transfection of *mafK* and *bach1b* repressed it. The activity of the *ctr1l* promoter increased when an increased amount of *nrf2a* was transfected, but decreased when an increased amount of *bach1b* was transfected. Higher amounts of Bach1a outcompeted Nrf2a and vice versa. The *ctr1l*-luc construct contained the 5′ upstream 1183-bp region that harbored two MAREs (at −6638 bp and at ~−5456 bp) and a 1558-bp basic promoter region (starting at position −1439 bp through to position ~119 bp) that was isolated from zebrafish genome DNA (see Materials and Methods). +, quantity of the plasmid transfected was 100 ng; ++, quantity of the plasmid transfected was 200 ng; ++++, quantity of the plasmid transfected was 400 ng. Treatment with hemin increased the activity of the *ctr1l* promoter in all cases. The red gradient scale indicates the increasing doses of Nrf2a, and the blue gradient scale indicates the increasing doses of Bach1b. Student’s *t*-tests were conducted. ^††^*P*<0.01, ^†††^*P*<0.001 compared with no hemin. ***P<0.001 compared with the group into which only *ctr1l*-luc and *mafK* were transfected (third group from the left on the graph). ^•••^*P*<0.001 for the indicated comparisons.

Sequence analysis showed that there are multiple MARE, or MARE-like sites, in the regulatory regions of all of the six zebrafish exocrine zymogens of interest (supplementary material Fig. S4). We isolated the 5′ regulatory fragments that contained MARE motifs from two exocrine zymogens – *ctr1l* (2741 bp) and *tryl* (2681 bp) – and fused them with a luciferase reporter gene. Subsequent *in vitro* cell transfection assays and treatment with hemin showed that the activities of these two gene promoters increased upon the application of an increasing amount of hemin (Fig. 5A; supplementary material Fig. S5A). Indeed, the promoter activities of these two zymogens required the presence of the integral MARE motif, as the *ctr1l* and *tryl* promoters with the mutated MARE sequences were inactive (Fig. 5B; supplementary material Fig. S5B). In particular, the activities of the *ctr1l* promoter appeared to increase as the number of MARE motifs was increased (Fig. 5B). These results suggest that heme activates the expression of the exocrine zymogens *ctr1l* and *tryl* in a dosage-dependent manner and that the activities of the two zymogen promoters require the integral MARE site.

### Roles of the Bach1b-MafK and Nrf2a-MafK heterodimers in heme-mediated regulation of exocrine peptidase precursor genes

To delineate the roles of the Bach1b-MafK and Nrf2a-MafK heterodimers in heme-mediated regulation of exocrine zymogens, we conducted assays that involved co-transfection of Mafk pathway genes and treatment with hemin. Co-transfection of *nrf2a* and *mafK* facilitated the activities of the two *ctr1l* and *tryl* gene promoters (Fig. 5C), whereas co-transfection of *bach1b* and *mafK* repressed them (supplementary material Fig. S5C). Moreover, higher doses of Nrf2a outcompeted Bach1b and activated the luciferase reporter constructs for the zymogens, whereas higher doses of Bach1b outcompeted Nrf2a and repressed them, even though the activating effects on these two zymogens, stimulated by higher doses of Nrf2a, were much greater than the repressive effects of higher doses of Bach1b (Fig. 5C; supplementary material Fig. S5C). It is noteworthy that treatment with 10 μM hemin for 1 hour was sufficient to enhance the activity of these two gene promoters in all cases – whether cells were transfected with *mafK* alone, or co-transfected in combination with *mafK* and *nrf2a* or *mafK* and *bach1b* (Fig. 5C; supplementary material Fig. S5C). These results show that the Nrf2a-MafK heterodimer activates these exocrine zymogens, whereas the Bach1b-MafK heterodimer represses them.

### The dynamic exchange of Bach1b and Nrf2a is mediated by heme

**Fig. 6.**
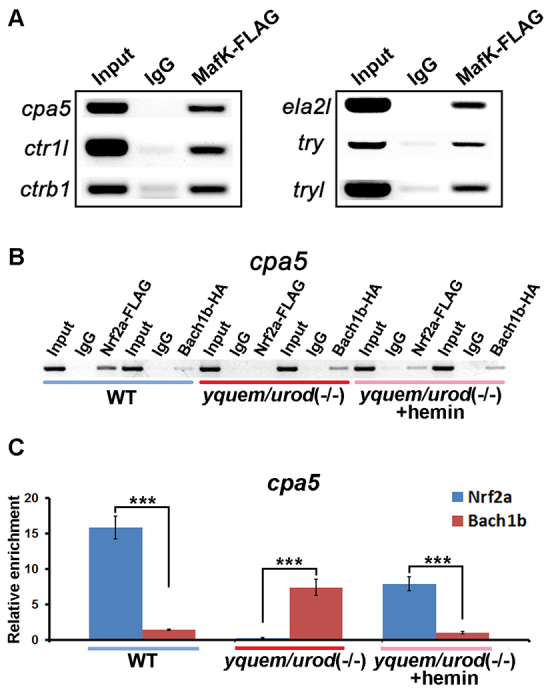
**ChIP assays.** Capped mRNAs encoding *mafK*-FLAG, *nrf2a*-FLAG or *bach1b*-HA were microinjected into one-cell stage embryos, antibodies against FLAG or HA were used to pull down complexes of the protein with the DNA, and the DNAs were eluted. Specific primers and the eluted DNAs were used in PCR analyses to amplify the DNA fragments that contained *cpa5* MARE sites, which were subsequently quantified by using qRT-PCR. (A) Electrophoresis analysis of the ChIP results showed that MafK bound to MARE-containing regulatory fragments of six zymogens. (B) Electrophoresis analysis of the *cpa5* ChIP assay. Approximately 2.6-fold more Nrf2a than Bach1b protein (quantified with ImageJ) was associated with *cpa5* promoters that harbored MARE sites in wild-type control larvae; whereas approximately 2.8-fold more Bach1b than Nrf2a protein (quantified with ImageJ) was associated with *cpa5* promoters that harbored MARE sites in heme-deficient *yquem/urod* (−/−) larvae. Moreover, treatment of the heme-deficient *yquem/urod* (−/−) larvae with hemin reversed the situation so that approximately 1.2-fold more Nrf2a than Bach1b protein (quantified with ImageJ) was associated with *cpa5* promoters that harbored MARE sites in hemin-treated *yquem/urod* (−/−) larvae. (C) qRT-PCR analysis of the *cpa5* ChIP assay, the results of which were consistent with the gel electrophoresis analysis in B.

Previous studies have shown that MafK can form a heterodimer with either Nrf2a or Bach1 (Takagi et al., 2004; Zenke-Kawasaki et al., 2007), and an electrophoretic mobility shift assay was used to show that zebrafish MafK binds to the MARE site (Takagi et al., 2004). To determine whether zebrafish MafK can bind to the MARE-harboring regulatory regions of zebrafish exocrine zymogens, we performed chromatin immunoprecipitation (ChIP) assays. The results showed that zebrafish MafK bound to the MARE-containing promoters of all six zebrafish exocrine zymogens (Fig. 6A). We also conducted ChIP assays to investigate exactly how heme employs Bach1b, Nrf2a and MafK to regulate these exocrine zymogens *in vivo*. The ChIP results showed that, in the wild-type control larvae with a normal level of heme, more Nrf2a than Bach1b interacted with the MARE-containing regulatory regions of the zymogens. Conversely, in the heme-deficient HEP larvae, more Bach1b than Nrf2a appeared to be associated with the MARE-containing regulatory regions of the zymogens. In particular, treatment of the HEP larvae with hemin reversed this situation so that more Nrf2a protein than Bach1b protein associated with the MARE-containing regulatory regions of the zymogens (Fig. 6B,C; supplementary material Fig. S6A–F). These data suggest that heme mediates the dynamic exchange of Bach1b and Nrf2a in their heterodimerization with MafK, which in turn exerts regulatory effects on the exocrine zymogens, in a manner similar that in which they regulate their other downstream genes (Ogawa et al., 2001; Sun et al., 2004; Marro et al., 2010).

## DISCUSSION

**Fig. 7.**
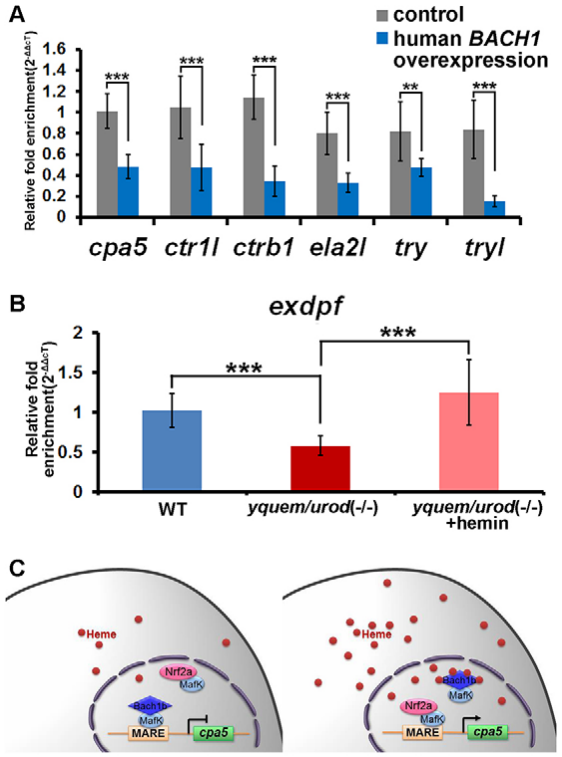
**The regulatory functions of BACH1 on exocrine zymogens are conserved, developmental delay of the exocrine pancreas in the HEP fish and a model for heme-mediated regulation of the exocrine peptidase precursor gene.** (A) Overexpression of human *BACH1* in zebrafish resulted in downregulation of the six peptidase precursor genes investigated. (B) Downregulation of *exdpf* (Jiang et al., 2008), an exocrine pancreas marker, in zebrafish *yquem/urod* (−/−), as determined by qRT-PCR analysis, the results of which are consistent with those achieved by using *in situ* hybridization (shown in supplementary material Fig. S7A,B). The primers used for the qRT-PCR analysis are listed in supplementary material Table S1. Student’s *t*-tests were conducted. **P<0.01, ***P<0.001. (C) A model for heme-mediated regulation of the exocrine peptidase precursor gene *cpa5* through the heterodimers Bach1b-MafK and Nrf2a-MafK. At low heme levels, the Bach1b-MafK heterodimer occupies MARE sites in the promoter of *cpa5* and represses its transcription (left panel); conversely, at higher heme levels, heme binds to Bach1b, stimulating the replacement of the Bach1b-MafK heterodimer with the Nrf2a-MafK heterodimer, activating the expression of *cpa5* (right panel).

Zebrafish possess duplicate genes *bach1a* and *bach1b* (Fig. 1B), and *nrf2a* and *nrf2b* (Timme-Laragy et al., 2012), which are derived from an ancient genome-wide duplication that occurred in teleost fish – including zebrafish (Aparicio et al., 2002; Jaillon et al., 2004; Kasahara et al., 2007). These two pairs of ancient duplicates appear to have evolved distinct functions through subfunctionalization (Postlethwait et al., 2004; Wang, 2008; Timme-Laragy et al., 2012). Here, our experiments, using overexpression and knockdown of *bach1b* and *nrf2a*, demonstrated the antagonism between Bach1b and Nrf2a in heme-mediated regulation of the zymogens Cpa5, Ctr1l, Ctrb1, Ela2l, Try and Tryl (Figs 3, 4; supplementary material Figs S2, S3). Previous mouse gene targeting studies have also shown the opposing functions of Bach1 and Nrf2 on the expression of mouse heme oxygenase 1 (*Ho-1*) – i.e. *Ho-1* is upregulated in *Bach1* knockout mice and downregulated in *Nrf2*-deficient cells (Sun et al., 2002). Also, our *in vitro* experiments showed that higher doses of *nrf2a* outcompete *bach1b* and thus enhance the expression of the target zymogens, whereas higher doses of *bach1b* outcompete *nrf2a* and repress expression of the zymogens examined here (Fig. 5C; supplementary material Fig. S5C). Moreover, our *in situ* hybridization experiments showed that *bach1b* and *nrf2a*, as well as *mafK*, are indeed expressed in the zebrafish exocrine pancreas (Fig. 2). However, whether the other two duplicates Bach1a and Nrf2b play roles in heme-mediated regulation of the six zymogens requires further investigation. Importantly, the regulatory functions of Bach1 on exocrine zymogens appear to be highly conserved, as evidenced by the fact that overexpression of human *BACH1* represses these six exocrine zymogens in zebrafish larvae (Fig. 7A).

The delay of the development of the exocrine pancreas in HEP fish does not play a substantial role in downregulation of the zymogens that were investigated here. Although both qRT-PCR and *in situ* hybridization experiments with the exocrine cell determination gene *exocrine differentiation and proliferation factor* (*expdf*) (Jiang et al., 2008) showed that there is, indeed, a degree of delay in the development of the exocrine pancreas in the HEP fish (Fig. 7B; supplementary material Fig. S7), the fact that treatment with hemin rapidly rescues the expression of the exocrine zymogens indicates the presence of sufficient acinar cells (Wang et al., 2007). We believe that heme deficiency is the major cause of the downregulation of the zymogens Cpa5, Ctr1l, Ctrb1, Ela2l, Try and Tryl in the HEP fish.

As zebrafish Bach1b contains HRMs with CP dipeptide (Fig. 1A), it mediates the regulatory role of heme in transcription of the zymogens investigated here (Wang et al., 2007). Heme binds to Bach1 through its CP-rich HRMs, leading to its Crm1-dependent nuclear export (Suzuki et al., 2004), and subsequently its ubiquitination and degradation (Zenke-Kawasaki et al., 2007). Consequently, Nrf2 heterodimerizes with MafK and activates the transcription of the downstream genes (Suzuki et al., 2004; Zenke-Kawasaki et al., 2007).

Our study reveals the roles of Bach1b, Nrf2a and MafK in heme-mediated regulation of exocrine peptidase precursor genes. Similar to regulation of other genes by the MafK pathway (Igarashi et al., 1998; Ogawa et al., 2001; Kitamuro et al., 2003; Brand et al., 2004; Sun et al., 2004; Marro et al., 2010), we observed that the Bach1b-MafK heterodimer represses the expression of the exocrine zymogens (Fig. 5C), whereas the Nrf2a-MafK heterodimer activates them (supplementary material Fig. S5C).

Our data indicate that heme does not only activate zymogens in a concentration-dependent manner (Fig. 5A; supplementary material Fig. S5A), but also that Bach1b dissociation from the MafK-occupied MARE sites is heme-dependent. In wild-type fish, more Nrf2a than Bach1b protein was associated with the MafK-occupied MARE sites in the regulatory regions of the zymogens. By contrast, in heme-deficient HEP fish, more Bach1b than Nrf2a protein was associated with the MafK-occupied MARE sites, which was reversed by treatment with hemin (Fig. 6B,C; supplementary material Fig. S6A–F). It appears that, through binding to Bach1b, heme induces the replacement of Bach1b with Nrf2a on the MafK-occupied MARE sites, switching from the repressive Bach1b-MafK heterodimer to the activating Nrf2a-MafK heterodimer (Fig. 6B,C; supplementary material Fig. S6A–F). Thus, through mediating the dynamic exchange of Bach1b and Nrf2a in the MafK-occupied MARE sites, heme plays a crucial role in the regulation of zymogen production.

Taken together, these data provide a molecular explanation of the frequently reported acute episodic abdominal pains with co-occurrences of nausea and vomiting in some porphyria individuals (Bickers, 1981; Pierach, 1982; Bonkovsky, 2005; Puy et al., 2010; Balwani and Desnick, 2012). In fact, treatment with hemin can effectively cure these symptoms, although the underlying mechanism for this kind of therapy has been elusive (Bickers, 1981; Pierach, 1982; Ryter and Tyrrell, 2000; Anderson et al., 2005; Bonkovsky, 2005; Solinas and Vajda, 2008; Cappellini et al., 2010; Puy et al., 2010; Siegesmund et al., 2010; Balwani and Desnick, 2012). Our study reveals why the HEP fish produce lower levels of zymogens and how hemin restores production of these proteins (Fig. 6B,C; supplementary material Fig. S6A–F). Because the roles of Bach1b, Nrf2a and MafK in heme-mediated regulation of exocrine zymogens appear to be highly conserved between zebrafish and human (Fig. 7A), we believe that the acute episodic abdominal pains in these porphyria individuals are likely to stem from decreased production of pancreatic zymogens. It is tempting to speculate that our study also provides a new therapeutic target for these porphyria individuals – for instance, the possibility of knocking down *BACH1* specifically in the exocrine pancreas to enhance zymogen levels (Fig. 7A), thereby, alleviating acute episodic abdominal pains.

In summary, we have revealed the antagonistic roles of the Nrf2a-MafK and Bach1b-MafK heterodimers in heme-mediated regulation of the exocrine pancreatic zymogens Cpa5, Ctr1l, Ctrb1, Ela2l, Try and Tryl. In conditions of heme deficiency, such as porphyria, the repressive Bach1b-MafK heterodimer prevails; and through its binding to Bach1b, hemin facilitates the formation of the activating Nrf2a-MafK heterodimer, restoring the expression of these zymogens in porphyria (Fig. 7C). These findings should provide novel targets for diagnosing and treating porphyria.

## MATERIALS AND METHODS

### Fish husbandry and embryo production

The Soochow University Animal Use and Care Committee approved all animal protocols. Zebrafish (*Danio rerio*) wild-type AB strain and the mutant line *yquem* (*yqe^tp61^*) were raised at the Soochow University Zebrafish Facility according to standard protocols (Westfield, 1995). Wild-type and mutant embryos were produced by pair mating, the embryos were then collected for RNA isolation and fixed for *in situ* hybridization experiments at the specified stages. Homozygous mutants (*yquem*/*urod*, *−/−*) were obtained by mating heterozygous fish (*yquem*/*urod*, *−/+*) and then identified by using an epifluorescent stereomicroscope (Leica M165 FC).

### RNA extraction and qRT-PCR

Total RNA was extracted using the TRIzol^®^ Reagent, according to the manufacturer’s instructions (Invitrogen). cDNA was synthesized by using reverse transcription with the M-MLV reverse transcription kit (Invitrogen), which was then used as the template for qRT-PCR analysis. qRT-PCR reactions were performed with the ABI StepOnePlus™ system, using SYBR^®^ Premix Ex Taq™ (TaKaRa) and the following thermal profile: 95°C for 3 minutes; 95°C for 10 seconds; 58°C for 30 seconds for 40 cycles. The primers that were used to amplify *cpa*, *ctr1l*, *ctrb1*, *ela2l*, *try*, *tryl* and *actb1* (as an internal control) are listed in supplementary material Table S1. Relative mRNA expression levels were quantified using the comparative *C*_t_(Δ*C*_t_) method and expressed as 2^−(ΔΔ*C*t)^. The calculations and statistical analyses were performed by using Microsoft Office Excel or SPSS10.0.1. Each PCR assay was performed on three biological samples.

### Generation of RNA probes

RNA probes for the six zymogens (*cpa5*, *ctr1l*, *ctrb1*, *ela2l*, *try* and *tryl*) and *exdpf* (Jiang et al., 2008) were generated, as previously described (Wang et al., 2007). The cDNA fragments for *bach1b* and *mafK* were amplified by using PCR and zebrafish larvae [96 hours post-fertilization (hpf)] cDNAs. These fragments were then cloned into the pEASY-T3 vector using the pEASY-T3 Cloning Kit (TransGen). A cDNA fragment of *nrf2a* was amplified, by using PCR, from pCS2*nrf2a*, a kind gift from Makodo Kobayashi (Takagi et al., 2004), and was then cloned into the pEASY-T3 vector. DIG-labeled RNA probes for *bach1b*, *nrf2a* and *mafK*, were produced using the DIG RNA Labeling Kit, and a fluorescein-labeled *try* probe was generated using the Fluorescein RNA Labeling Mix, according to the manufacturer’s instructions (Roche). Primers for *bach1b*, *nrf2a* and *mafK* are listed in supplementary material Table S1.

### Whole-mount *in situ* hybridization analysis of gene expression

Whole-mount *in situ* hybridization experiments were conducted as previously described (Wang et al., 2007). Briefly, 10–15 larvae were used for each *in situ* hybridization experiment. At least three independent experiments were conducted for each gene by using an antisense probe. Double *in situ* hybridization assays were performed by using digoxigenin and fluorescein-labeled probes, and developed with the chromagenic substrates 5-bromo-4-chloro-3-indolyl phosphate (BCIP) and nitroblue tetrazolium, and Fast Red. Following *in situ* hybridization staining, selected larvae were rehydrated and embedded in 30% sucrose at 4°C overnight for cryostat sectioning. Sectioning was performed with a Leica CM 1850 cryostat. Stained larvae and sections were photographed using a stereomicroscope (Leica M165 FC) with a digital camera, and the images were analyzed by using ImageJ (National Institutes of Health) and Adobe Photoshop.

### Construction of the expression vectors

The full-length *bach1b* cDNA sequence was isolated by using the SMARTer™ RACE cDNA Amplification Kit, according to the manufacturer’s instructions (Clontech) and deposited in NCBI (accession number KJ420533). The largest *bach1b* open reading frame (ORF) of 1899 bp encodes a peptide of 632 amino acids (Fig. 1A), which was amplified by using PCR of zebrafish larval cDNAs (120 hpf) as template. This ORF was then cloned into the pEASY-T3 vector, and the final construct was named p*bach1b*-T3. The plasmids pcDNA3.1*mafK*-FLAG, pcDNA3.1*nrf2a*-FLAG and pcDNA*bach1b*-HA were constructed by inserting the cDNA sequences corresponding to the FLAG-tag (DYKDDDDK) or HA-tag (YPYDVPDYA) before the stop codon TAA sequence at the 3′ ends of the *mafK*, *nrf2a* and *bach1b* ORF cDNAs. The primers used for *bach1b* ORF cloning are listed in supplementary material Table S1.

### Overexpression and knockdown of *bach1b* and *nrf2a*

The plasmids p*bach1b*-T3 and pCS2*nrf2*a were linearized and used as templates for generating capped mRNAs with the mMESSAGE mMACHINE^®^ Kit according to the manufacturer’s instructions (Ambion). The SPL MO (5′-CCTTTGATTGTGTCTTTACCTCATC-3′) and ATG MO (5′-TGACTTTGAGCTTTCCACCGACATC-3′) against *bach1b*, and the *nrf2a* ATG MO (5′-CATTTCAATCTCCATCATGTCTCAG-3′) (Kobayashi et al., 2002; Kobayashi et al., 2009; Wang and Gallagher, 2013) were purchased from Gene Tools. A mixture of *bach1b* (or *nrf2a*) capped mRNA and Tris-HCl (0.01 M, pH 7.0), or MO against *bach1b* (or *nrf2a*) dissolved in Danieau buffer [58 mM NaCl, 0.7 mM KCl, 0.4 mM MgSO_4_, 0.6 mM Ca(NO_3_)_2_, 5.0 mM HEPES (pH 7.6)] was microinjected into one-cell stage zebrafish embryos. Microinjection controls with the vehicle solution, lacking mRNA or MO, were also performed. Reverse transcription PCR showed that the *bach1b* SPL MO effectively blocked the splicing of intron 2 (supplementary material Fig. S1A,B). The ATG MO knocked down *bach1b* with equal efficacy to that of the SPL MO (supplementary material Fig. S1C). The efficacy of the *nrf2a* ATG MO has been demonstrated previously (Kobayashi et al., 2002; Kobayashi et al., 2009; Wang and Gallagher, 2013). Total RNA was isolated from 30–50 larvae at 84 hpf and reverse transcribed to cDNAs for qRT-PCR analysis. The corresponding larvae were also fixed for *in situ* hybridization experiments. At least three independent replicates were performed for each experiment. The primers that were used for the *bach1b* intron 1 splicing analysis are listed in supplementary material Table S1.

### Construction of the luciferase reporter vectors

The 5′ promoter regions of these six zymogens all contain multiple MARE (Maf recognition element) or MARE like sites (supplementary material Fig. S1). Among them, the *ctr1l* and *tryl* promoter fragments containing MARE sites were PCR amplified from the genomic DNA, first cloned into pMD19-T simple vector (TaKaRa), and then subcloned into luciferase reporter vector pGL3-Basic. The *ctr1l*-luc construct contained the 5′ upstream 1183-bp region that harbored two MAREs (starting at position −6638 bp through to position −5456 bp) and a 1558-bp basic promoter region (starting at position −1439 bp through to position 119 bp), whereas the *tryl*-luc construct contains 1604 bp harboring one MARE (−6371 bp to –4768 bp) and a 1077-bp basic promoter region (−886 bp to 191 bp). The MARE binding sites also were mutated using the site-directed mutagenesis kit according to the manufacturer’s instructions (TaKaRa). The corresponding primers are listed in supplementary material Table S1.

### Cell culture, cell transfection and luciferase assay

The NIH3T3 and 293T cell lines were cultured in Dulbecco’s modified Eagle medium (DMEM; high glucose, Invitrogen). The medium was supplemented with 10% heat-inactivated low-endotoxin fetal bovine serum (FBS; Invitrogen), 100 μg/ml penicillin and 100 μg/ml streptomycin. Cells were maintained at 37°C under 5% CO_2_ inside an incubator. NIH3T3 or 293T cells were plated at 70% confluence in 24-well plates and incubated overnight. Transfections were performed using Lipofectamine 2000, according to the manufacturer’s instructions (Invitrogen). Five hours after transfection, the cells were treated with the indicated concentrations of hemin. Cells were lysed in passive lysis buffer (Promega) at the indicated timepoints, and cellular extracts were analyzed for luciferase activity using the Dual-Luciferase-Reporter Assay System (Promega) and a Luminoskan Ascent Microplate Luminometer (Thermo Scientific). The pCS2*mafK*, pCS2*nrf2a* and pcDNA3.1*bach1b*-HA constructs were co-transfected with luciferase reporter constructs. Control cells were transfected with the pGL3-Basic vector, as well as a control plasmid containing the *Renilla* gene under the control of the HSV-TK promoter. Transfection efficiencies were normalized to the activity of a *Renilla* luciferase expression plasmid pRL-TK. Each experiment was performed three times.

### ChIP assays

The ChIP analyses were performed using the ChIP Assay Kit, according to the manufacturer’s instructions (Millipore). Briefly, the embryos that had been microinjected with capped mRNAs of *mafK*-FLAG, *nrf2a*-FLAG or *bach1b*-HA were fixed at 96 hpf by using formaldehyde (final concentration at 1%) for 20 minutes at room temperature. The larvae then were lysed using SDS lysis buffer. The chromatin lysates were sonicated to 200–1000 bp DNA fragments. Monoclonal antibodies against the FLAG and HA tags (EarthOx) were used for immunoprecipitation. After extensive washing, the DNAs were eluted using elution buffer (1% SDS, 0.5 M NaHCO_3_) and the eluted DNA was analyzed by using PCR. The PCR products were quantified by using qRT-PCR (Invitrogen). Relative enrichment was calculated as the difference between the specific antibody and normal IgG signals that had been normalized to the respective input signals. The primers flanking the MARE sites in the upstream regions of the six exocrine zymogens are listed in supplementary material Table S1.

### Treatment with hemin

Hemin (Sigma) was dissolved in 0.2 ml of 1 N NaOH and then 1 ml of 0.2 M Tris-HCl (pH 8), distilled and deionized water was added to yield the desired concentration. The pH was adjusted to 7.8 by using 1 N HCl. Treatment with hemin was performed as described previously (Wang et al., 2007).

## Supplementary Material

Supplementary Material

## References

[b1-0070837] Amores A. Catchen J. Ferrara A. Fontenot Q. Postlethwait J. H. (2011). Genome evolution and meiotic maps by massively parallel DNA sequencing: spotted gar, an outgroup for the teleost genome duplication. Genetics 188, 799–808 21828280 10.1534/genetics.111.127324 PMC3176089

[b2-0070837] Anderson K. E. Bloomer J. R. Bonkovsky H. L. Kushner J. P. Pierach C. A. Pimstone N. R. Desnick R. J. (2005). Recommendations for the diagnosis and treatment of the acute porphyrias. Ann. Intern. Med. 142, 439–450 15767622 10.7326/0003-4819-142-6-200503150-00010

[b3-0070837] Aparicio S. Chapman J. Stupka E. Putnam N. Chia J. M. Dehal P. Christoffels A. Rash S. Hoon S. Smit A. (2002). Whole-genome shotgun assembly and analysis of the genome of Fugu rubripes. Science 297, 1301–1310 12142439 10.1126/science.1072104

[b4-0070837] Balwani M. Desnick R. J. (2012). The porphyrias: advances in diagnosis and treatment. Blood 120, 4496–4504 22791288 10.1182/blood-2012-05-423186 PMC3512229

[b5-0070837] Bickers D. R. (1981). Treatment of the porphyrias: mechanisms of action. J. Invest. Dermatol. 77, 107–113 7252241 10.1111/1523-1747.ep12479285

[b6-0070837] Blank V. (2008). Small Maf proteins in mammalian gene control: mere dimerization partners or dynamic transcriptional regulators? J. Mol. Biol. 376, 913–925 18201722 10.1016/j.jmb.2007.11.074

[b7-0070837] Bonkovsky H. L. (2005). Neurovisceral porphyrias: what a hematologist needs to know. Hematology (Am. Soc. Hematol. Educ. Program) 2005, 24–30 16304355 10.1182/asheducation-2005.1.24

[b8-0070837] Brand M. Ranish J. A. Kummer N. T. Hamilton J. Igarashi K. Francastel C. Chi T. H. Crabtree G. R. Aebersold R. Groudine M. (2004). Dynamic changes in transcription factor complexes during erythroid differentiation revealed by quantitative proteomics. Nat. Struct. Mol. Biol. 11, 73–80 14718926 10.1038/nsmb713

[b9-0070837] Cappellini M. D. Brancaleoni V. Graziadei G. Tavazzi D. Di Pierro E. (2010). Porphyrias at a glance: diagnosis and treatment. Intern. Emerg. Med. 5 Suppl. 1, S73–S80 20865478 10.1007/s11739-010-0449-7

[b10-0070837] Hedstrom L. (2002). Serine protease mechanism and specificity. Chem. Rev. 102, 4501–4524 12475199 10.1021/cr000033x

[b11-0070837] Igarashi K. Sun J. (2006). The heme-Bach1 pathway in the regulation of oxidative stress response and erythroid differentiation. Antioxid. Redox Signal. 8, 107–118 16487043 10.1089/ars.2006.8.107

[b12-0070837] Igarashi K. Hoshino H. Muto A. Suwabe N. Nishikawa S. Nakauchi H. Yamamoto M. (1998). Multivalent DNA binding complex generated by small Maf and Bach1 as a possible biochemical basis for beta-globin locus control region complex. J. Biol. Chem. 273, 11783–11790 9565602 10.1074/jbc.273.19.11783

[b13-0070837] Jaillon O. Aury J. M. Brunet F. Petit J. L. Stange-Thomann N. Mauceli E. Bouneau L. Fischer C. Ozouf-Costaz C. Bernot A. (2004). Genome duplication in the teleost fish Tetraodon nigroviridis reveals the early vertebrate proto-karyotype. Nature 431, 946–957 15496914 10.1038/nature03025

[b14-0070837] Jiang Z. Song J. Qi F. Xiao A. An X. Liu N. A. Zhu Z. Zhang B. Lin S. (2008). Exdpf is a key regulator of exocrine pancreas development controlled by retinoic acid and ptf1a in zebrafish. PLoS Biol. 6, e293 19067490 10.1371/journal.pbio.0060293 PMC2586380

[b15-0070837] Kappas A. Sassa S. Galbraith R. A. Nordmann Y. (1995). The porphyrias. In The Metabolic Basis of Inherited Diseases (ed. Scribe C. R. Beaudet A. L. Sly W. S.), pp. 2103–2159 New York, NY: McGraw-Hill

[b16-0070837] Kasahara M. Naruse K. Sasaki S. Nakatani Y. Qu W. Ahsan B. Yamada T. Nagayasu Y. Doi K. Kasai Y. (2007). The medaka draft genome and insights into vertebrate genome evolution. Nature 447, 714–719 17554307 10.1038/nature05846

[b17-0070837] Kitamuro T. Takahashi K. Ogawa K. Udono-Fujimori R. Takeda K. Furuyama K. Nakayama M. Sun J. Fujita H. Hida W. (2003). Bach1 functions as a hypoxia-inducible repressor for the heme oxygenase-1 gene in human cells. J. Biol. Chem. 278, 9125–9133 12511571 10.1074/jbc.M209939200

[b18-0070837] Kobayashi M. Yamamoto M. (2006). Nrf2-Keap1 regulation of cellular defense mechanisms against electrophiles and reactive oxygen species. Adv. Enzyme Regul. 46, 113–140 16887173 10.1016/j.advenzreg.2006.01.007

[b19-0070837] Kobayashi M. Itoh K. Suzuki T. Osanai H. Nishikawa K. Katoh Y. Takagi Y. Yamamoto M. (2002). Identification of the interactive interface and phylogenic conservation of the Nrf2-Keap1 system. Genes Cells 7, 807–820 12167159 10.1046/j.1365-2443.2002.00561.x

[b20-0070837] Kobayashi M. Li L. Iwamoto N. Nakajima-Takagi Y. Kaneko H. Nakayama Y. Eguchi M. Wada Y. Kumagai Y. Yamamoto M. (2009). The antioxidant defense system Keap1-Nrf2 comprises a multiple sensing mechanism for responding to a wide range of chemical compounds. Mol. Cell. Biol. 29, 493–502 19001094 10.1128/MCB.01080-08 PMC2612520

[b21-0070837] Marro S. Chiabrando D. Messana E. Stolte J. Turco E. Tolosano E. Muckenthaler M. U. (2010). Heme controls ferroportin1 (FPN1) transcription involving Bach1, Nrf2 and a MARE/ARE sequence motif at position -7007 of the FPN1 promoter. Haematologica 95, 1261–1268 20179090 10.3324/haematol.2009.020123 PMC2913073

[b22-0070837] Mense S. M. Zhang L. (2006). Heme: a versatile signaling molecule controlling the activities of diverse regulators ranging from transcription factors to MAP kinases. Cell Res. 16, 681–692 16894358 10.1038/sj.cr.7310086

[b23-0070837] Ogawa K. Sun J. Taketani S. Nakajima O. Nishitani C. Sassa S. Hayashi N. Yamamoto M. Shibahara S. Fujita H. (2001). Heme mediates derepression of Maf recognition element through direct binding to transcription repressor Bach1. EMBO J. 20, 2835–2843 11387216 10.1093/emboj/20.11.2835 PMC125477

[b24-0070837] Padmanaban G. Venkateswar V. Rangarajan P. N. (1989). Haem as a multifunctional regulator. Trends Biochem. Sci. 14, 492–496 2696180 10.1016/0968-0004(89)90182-5

[b25-0070837] Pierach C. A. (1982). Hematin therapy for the porphyric attack. Semin. Liver Dis. 2, 125–131 6753162 10.1055/s-2008-1040702

[b26-0070837] Postlethwait J. Amores A. Cresko W. Singer A. Yan Y. L. (2004). Subfunction partitioning, the teleost radiation and the annotation of the human genome. Trends Genet. 20, 481–490 15363902 10.1016/j.tig.2004.08.001

[b27-0070837] Pratt S. J. Drejer A. Foott H. Barut B. Brownlie A. Postlethwait J. Kato Y. Yamamoto M. Zon L. I. (2002). Isolation and characterization of zebrafish NFE2. Physiol. Genomics 11, 91–98 12388799 10.1152/physiolgenomics.00112.2001

[b28-0070837] Puy H. Gouya L. Deybach J. C. (2010). Porphyrias. Lancet 375, 924–937 20226990 10.1016/S0140-6736(09)61925-5

[b29-0070837] Ryter S. W. Tyrrell R. M. (2000). The heme synthesis and degradation pathways: role in oxidant sensitivity. Heme oxygenase has both pro- and antioxidant properties. Free Radic. Biol. Med. 28, 289–309 11281297 10.1016/s0891-5849(99)00223-3

[b30-0070837] Sassa S. Nagai T. (1996). The role of heme in gene expression. Int. J. Hematol. 63, 167–178 8936331 10.1016/0925-5710(96)00449-5

[b31-0070837] Siegesmund M. van Tuyll van Serooskerken A. M. Poblete-Gutiérrez P. Frank J. (2010). The acute hepatic porphyrias: current status and future challenges. Best Pract. Res. Clin. Gastroenterol. 24, 593–605 20955962 10.1016/j.bpg.2010.08.010

[b32-0070837] Solinas C. Vajda F. J. (2008). Neurological complications of porphyria. J. Clin. Neurosci. 15, 263–268 18187328 10.1016/j.jocn.2006.11.015

[b33-0070837] Sun J. Hoshino H. Takaku K. Nakajima O. Muto A. Suzuki H. Tashiro S. Takahashi S. Shibahara S. Alam J. (2002). Hemoprotein Bach1 regulates enhancer availability of heme oxygenase-1 gene. EMBO J. 21, 5216–5224 12356737 10.1093/emboj/cdf516 PMC129038

[b34-0070837] Sun J. Brand M. Zenke Y. Tashiro S. Groudine M. Igarashi K. (2004). Heme regulates the dynamic exchange of Bach1 and NF-E2-related factors in the Maf transcription factor network. Proc. Natl. Acad. Sci. USA 101, 1461–1466 14747657 10.1073/pnas.0308083100 PMC341742

[b35-0070837] Suzuki H. Tashiro S. Hira S. Sun J. Yamazaki C. Zenke Y. Ikeda-Saito M. Yoshida M. Igarashi K. (2004). Heme regulates gene expression by triggering Crm1-dependent nuclear export of Bach1. EMBO J. 23, 2544–2553 15175654 10.1038/sj.emboj.7600248 PMC449764

[b36-0070837] Takagi Y. Kobayashi M. Li L. Suzuki T. Nishikawa K. Yamamoto M. (2004). MafT, a new member of the small Maf protein family in zebrafish. Biochem. Biophys. Res. Commun. 320, 62–69 15207702 10.1016/j.bbrc.2004.05.131

[b37-0070837] Tamura K. Peterson D. Peterson N. Stecher G. Nei M. Kumar S. (2011). MEGA5: molecular evolutionary genetics analysis using maximum likelihood, evolutionary distance, and maximum parsimony methods. Mol. Biol. Evol. 28, 2731–2739 21546353 10.1093/molbev/msr121 PMC3203626

[b38-0070837] Timme-Laragy A. R. Karchner S. I. Franks D. G. Jenny M. J. Harbeitner R. C. Goldstone J. V. McArthur A. G. Hahn M. E. (2012). Nrf2b, novel zebrafish paralog of oxidant-responsive transcription factor NF-E2-related factor 2 (NRF2). J. Biol. Chem. 287, 4609–4627 22174413 10.1074/jbc.M111.260125 PMC3281635

[b39-0070837] Toki T. Katsuoka F. Kanezaki R. Xu G. Kurotaki H. Sun J. Kamio T. Watanabe S. Tandai S. Terui K. (2005). Transgenic expression of BACH1 transcription factor results in megakaryocytic impairment. Blood 105, 3100–3108 15613547 10.1182/blood-2004-07-2826

[b40-0070837] Wang H. (2008). Comparative analysis of period genes in teleost fish genomes. J. Mol. Evol. 67, 29–40 18535754 10.1007/s00239-008-9121-5

[b41-0070837] Wang L. Gallagher E. P. (2013). Role of Nrf2 antioxidant defense in mitigating cadmium-induced oxidative stress in the olfactory system of zebrafish. Toxicol. Appl. Pharmacol. 266, 177–186 23174481 10.1016/j.taap.2012.11.010

[b42-0070837] Wang H. Long Q. Marty S. D. Sassa S. Lin S. (1998). A zebrafish model for hepatoerythropoietic porphyria. Nat. Genet. 20, 239–243 9806541 10.1038/3041

[b43-0070837] Wang H. Zhou Q. Kesinger J. W. Norris C. Valdez C. (2007). Heme regulates exocrine peptidase precursor genes in zebrafish. Exp. Biol. Med. (Maywood) 232, 1170–1180 17895525 10.3181/0703-RM-77

[b44-0070837] Westfield M. (1995). The Zebrafish Book. Eugene, OR: The University of Oregon Press

[b45-0070837] Zenke-Kawasaki Y. Dohi Y. Katoh Y. Ikura T. Ikura M. Asahara T. Tokunaga F. Iwai K. Igarashi K. (2007). Heme induces ubiquitination and degradation of the transcription factor Bach1. Mol. Cell. Biol. 27, 6962–6971 17682061 10.1128/MCB.02415-06 PMC2099246

