# Supplementary material for: Heme acts through the Bach1b/Nrf2a-MafK pathway to regulate exocrine peptidase precursor genes in porphyric zebrafish
Source: Dis Model Mech. 2014 Mar 20;7(7):837–45. doi: 10.1242/dmm.014951 (PMC4073273; doi:10.1242/dmm.014951)
Supplement: Supplementary Material [file supp_7_7_837__index.html]

Heme acts through the Bach1b/Nrf2a-MafK pathway to regulate exocrine peptidase precursor genes in porphyric zebrafish — Supplementary Material 

# Heme acts through the Bach1b/Nrf2a-MafK pathway to regulate exocrine peptidase precursor genes in porphyric zebrafish

## DMM014951 Supplementary Material

**Files in this Data Supplement:**

- **Supplementary Material**
