# Supplementary material for: Heme acts through the Bach1b/Nrf2a-MafK pathway to regulate exocrine peptidase precursor genes in porphyric zebrafish
Source: Dis Model Mech. 2014 Mar 20;7(7):837–45. doi: 10.1242/dmm.014951 (PMC4073273; doi:10.1242/dmm.014951)
Supplement: Supplementary Material [file supp_7.7.837_DMM014951.pdf]

Supplementary Materials

Zhang et al. Heme regulation of exocrine pancreatic zymogens

Supplementary tables:

7 **Table S1. List of primers**

gene	Forward primer	Reverse primer	Accession no.	note
cpa5 qRT-PCR	TGGAGGCACTATTGACTGGA	GATTGGCTGGCAGAATAAAA	ENSDART00000034377	qRT-PCR
ctr1l qRT-PCR	AGCATCCCATCTGGAACTCG	CTGGACTCAGAAGAGGCAAT	ENSDART00000099425	qRT-PCR
ctrb1 qRT-PCR	TCGCCGAGACCAACGACAAC	GCTGGAGCAGGGCAGGAGTA	ENSDART00000037346	qRT-PCR
ela2l qRT-PCR	CTGAGCCAGGAGGAGAACGG	TCAGGGCGATGTCATTACGG	ENSDART00000079293	qRT-PCR
try qRT-PCR	CTGATGTGCCTGAATGCTCC	CCTTGCCTCCCTCCATAAAA	ENSDART00000077661	qRT-PCR
tryl qRT-PCR	GAATCATCCTGGTGTTTACG	TATTTGGAGGCTGTATGTGA	ENSDART00000148846	qRT-PCR
exdpf qRT-PCR	GTTTACCCGACCAACCTT	CATTTTCCCCGTCCTCTA	ENSDART00000032996	qRT-PCR
actb1 qRT-PCR	ACGAACGACCAACCTAAACCTCT	TTAGACAACTACCTCCCTTTCC	ENSDART00000054987	qRT-PCR
bach1b RNA probe	TTGCCTCACAGTAAGAAGGT	GTAGTAAACGCTGCTCCATC	ENSDART00000047541	probe making
nrf2a RNA probe	GGAGGAGATGGAAGGAAGTC	TAAGGCGAGGAACTAGGAAA	ENSDART00000062854	probe making
mafK RNA probe	TTGTCAAGTCTGCCAATCAC	CACCAAGAACAAAGAGCAAC	ENSDART00000132619	probe making
bach1b 5'-RACE	CGAACCAGTGACCTTCTTACTGTGAGGCAA		ENSDART00000047541	5'-RACE
bach1b 3'-RACE	TGTTGTTTTAGATGGAGCAGCGTTTACTAC		ENSDART00000047541	3'-RACE
bach1b ORF	ACTCAAGATGTCGGTGGAAA	CAGTGAAAGGCACTGTAAAT		*bach1b CDS cloning*
bach1b intron1	CACCCGAAAGCATTGTCATC	GGAGCAGGAAATCAAAGCAG	ENSDARG00000002196	*bach1b* intron1
ctr1l-MARE	CGGTACCAATTGAATGCATTTATGAAGAAACATAG	TGCTAGCCCCTACCTCTAAACCCAACCC	ENSDARG00000068680	Promoter cloning
ctr1l-Basic	TGCTAGCCTGAAAATGTTCCCACGACCC	TCTCGAGAAAAGCATTATCTGAAGTATACCCATCT	ENSDARG00000068680	Promoter cloning
tryl-MARE	CGGTACCTTTTTGAGTTTTGTATTTTTGGATATTG	TGCTAGCGGCAGCAGTTGACCTAAAATGTG	ENSDARG00000079274	Promoter cloning
tryl-Basic	TGCTAGCGCCATAACTGAGACCACCTGA	TCTCGAGAGGGCTGGGAATTAGGCTGACACTC	ENSDARG00000079274	Promoter cloning
ctr1l-MARE1	AAAAAAAAACATTGACCGAACCTAAA	TACAGTAATGAGCTGTGATATTTGGA	ENSDARG00000068680	Promoter cloning
ctr1l-MARE2	TTTTTTGTTGACCGAACCTAAATCGA	TTTTAACCCAAGTACTATTGTTTAC	ENSDARG00000	Promoter

			068680	cloning
tryl-MARE1	GTATGTAATGACCGAAGTAATGAAAT	AAAAATGGTGAACTAGTTGTCAAAAT	ENSDARG00000079274	Promoter cloning
cpa5 ChIP	GCGCATCTCCTGTGCTAAAC	TGCAATACCACAGTGAGCCT	ENSDARG00000021339	ChIP analysis
ctr1l ChIP	TAATGTGCCACAGCCATTAA	GCAGGGGTCTTTGGGTTGAT	ENSDARG00000068680	ChIP analysis
ctrb1 ChIP	TATGCAGAGCCACAGATATT	GTTGACTGAAGGAAAACAGA	ENSDARG00000090428	ChIP analysis
ela2l ChIP	AACAACATGATAGGGCAAGA	CCTCAACTGGAAGATGTCTC	ENSDARG00000056765	ChIP analysis
try ChIP	GCACAGGGTCCTCACAAC	AGCGGAATGAATCACCAACT	ENSDARG00000042993	ChIP analysis
tryl ChIP	AACGATTCCTTGGTTTTGCC	GAAACCCAGCACCATTCTCC	ENSDARG00000079274	ChIP analysis
Hs-BACH1 ORF	ATGTCTCTGAGTGAGAACTCGGTTT	TTACTCATCAGTAGTACATTTATCA	ENST00000286800	*Hs-BACH1* ORF cloning

Table. S2. *Bach1* genes used in the phylogenetic analysis*

Species	Gene name	Ensembl Gene ID	Transcript length (bps)	Peptide length (aa)
Homo sapiens	*BACH1*	ENSG00000156273	5669	736
Mus musculus	*Bach1*	ENSMUSG00000025612	5867	739
Rattus norvegicus	*Bach1*	ENSRNOG00000001582	3232	739
Xenopus tropicalis	*bach1*	ENSXETG00000020266	5689	720
Tetraodon nigroviridis	*bach1b*	ENSTNIG00000001421	1824	608
Tetraodon nigroviridis	*bach1a*	ENSTNIG00000019024	1719	573
Takifugu rubripes	*bach1b*	ENSTRUG00000009007	1712	564
Takifugu rubripes	*bach1a*	ENSTRUG00000014936	1776	592
Oreochromis niloticus	*bach1a*	ENSONIG00000005635	1770	590
Oreochromis niloticus	*bach1b*	ENSONIG00000004708	1872	624
Oryzias latipes	*bach1a*	ENSORLG00000003339	1727	548
Oryzias latipes	*bach1b*	ENSORLG00000004475	2052	684
Gasterosteus aculeatus	*bach1a*	ENSGACG00000011798	1758	586
Danio rerio	*bach1a*	ENSDARG00000062553	6074	676
Danio rerio	*bach1b*	ENSDARG00000002196	2832	632
Ciona intestinalis	*bach*	ENSCING00000006014	2436	508
Ciona savignyi	*bach*	ENSCSAVG00000006130	2062	651

* Note: All information is from ensembl (http://www.ensembl.org/index.html).

Supplementary Fig. legends:

Fig. S1. Efficacy of the *bach1b* SPL MO and the similar knockdown effect by the *bach1b* ATG MO. (A) Specific primers flanking the *bach1b* intron 2 splicing site were used to assess the efficacy of *bach1b* SPL MO. (B) In wild-type embryos, only the 183bp fragment was amplified (line 1), while in SPL MO-injected embryos (line 2), an additional 2, 205bp fragment also was amplified. DNA sequencing analysis confirmed that part of the 2, 205bp fragment indeed were from the *bach1b* intron 2 sequences, and a premature stop codon resulted in a short peptide of only 88 amino acids. (C) Effective knockdown *bach1b* by the ATG MO, as shown by significant upregulation of these six zymogens in the *bach1b* ATG morphant.

Fig. S2. Exocrine pancreas-specific downregulation or upregulation of *ctr1l*, *ctrb1, ela2l, try* and *tryl* resulted from overexpression or knockdown of *bach1b* and *nrf2a*, revealed by *in situ* hybridization. Representative images of *in situ* hybridization staining show that upregulation or downregulation of *ctr1l*, *ctrb1, ela2l, try* and *tryl* specifically in the zebrafish exocrine pancreas. Downregulation of *ctr1l*, *ctrb1, ela2l, try* and *tryl* specifically in the exocrine pancreas resulted from *bach1b* overexpression and *nrf2a* knockdown, and upregulation of *ctr1l*, *ctrb1, ela2l, try* and *tryl* specifically in the exocrine pancreas resulted from *nrf2a* overexpression and *bach1b* knockdown. Dorsal view, anterior to left. All larvae were 84 HPF.

Fig. S3. (A) Mean optic densities of *in situ* hybridization staining of a group of larvae (10-15 each) for *ctr1l*, *ctrb1, ela2l, try* and *tryl* from the overexpression or knockdown of *bach1a* and *nrf2a* corresponding to Fig. S2, quantified with NIH ImageJ. Statistical significance of difference between means was determined by one-way ANOVA and Tukey's multiple comparison test (n=12) with SPSS10.0.1. * P<0.05, ** P<0.01, *** P<0.001. (B) Morphant phenotypes for *ctr1l*, *ctrb1, ela2l, try* and *tryl* from the overexpression or knockdown of *bach1a* and *nrf2a*. The same method as described in the Fig. 4E legend was used to classify the 'strong', 'medium' and 'weak' categories of the morphant phenotypes. For each group, more than 50% of larvae display 'strong' and 'medium' effects for these five peptidase precursor genes resulted from knockdown or overexpression experiments.

Fig. S4. Schematic diagram of MARE (TGCTGA（C/G）TCAGCA) or MARE like (CTGAN/NTCAG) sites in the 5' regulatory regions of these six peptidase precursor genes. All sequences were downloaded from ensembl (http://www.ensembl.org/index.html).

Fig. S5. MARE-dependent regulation of the *tryl* promoter activity *by* heme, Bach1b and Nrf2a. (A) Activities of the *tryl* promoter increase with increasing the amount of hemin used. (B) The *tryl* promoter's activities require the presence of the MARE site, and the *tryl* promoter with MARE displays stronger activities than that with mutated MARE. (C) Co-transfection of *mafK* and *nrf2a* enhances the *tryl* promoters' activities, while co-transfection of *mafK* and *bach1b* represses *tryl*

promoters' activities. Higher concentrations of Bach1a are able to outcompete Nrf2a, *vice visa*. Activities of the *tryl* promoter increase with increasing the concentration of *nrf2a* transfected, but decrease with increasing the concentration of *bach1b* plasmid transfected. The *tryl*-luc construct contains the 5' upstream 1, 604bp harboring 1 MARE (-6, 371bp ~ -4, 768bp) and 1, 077bp basic promoter region (-886bp ~191bp) isolated from zebrafish genome DNA (See MATERIALS AND METHODS). +, quantity of plasmid transfected is 100ng, ++, quantity of plasmid transfected is 200ng, and ++++, quantity of plasmid transfected is 400ng. Hemin treatment can increase the activities of the *tryl* promoter in all cases. Student's *t* tests were conducted. ††$P<0.01$, †††$P<0.001$ compared with no hemin. ***$P<0.001$ compared with line 3, in which group only *tryl*-luc and *MafK* were added. •••$P<0.001$.

Fig. S6. ChIP analyses with *ctr1l, ctrb1, ela2l, try* and *tryl*. (A) Electrophoresis analyses of ChIP results with *ctr1l*, *ctrb1, ela2l, try* and *tryl*. Much more Nrf2a proteins were shown to be associated with these peptidase precursor gene promoters harboring MARE sites than Bach1b in wild type control larvae; while much more Bach1b proteins were shown to be associated with these promoters harboring MARE sites than Nrf2a in heme-deficient *yquem/urod* (-/-) larvae. Moreover, treatment of the heme-deficient *yquem/urod* (-/-) larvae with hemin can reverse the situation so that more Nrf2a proteins were associated with these promoter harboring MARE sites in hemin-treated *yquem/urod* (-/-) larvae than Bach1b. qRT-PCR analyses of the ChIP results with *ctr1l* (B), *ctrb1* (C), *ela2l* (D), *try* (E) and *tryl* (F). The results of these

qRT-PCR analyses are consistent with the gel electrophoresis analyses shown in Fig. S6A. Student's *t* tests were conducted. ***P<0.001.

Fig. S7. (A) Representative images of *in situ* hybridization staining indicate downregulation of *exdpf* in zebrafish *yquem/urod* (-/-). Despite the expression of *exdpf* elsewhere in larvae, it is the exocrine pancreatic expression that is most affected in the mutant fish. Dorsal view, anterior to left. (B) Mean optic densities of *in situ* hybridization staining of a group of larvae (10-12 each) corresponding to Fig. S7A quantified with ImageJ. Student's *t* tests were conducted. * P<0.05. These *in situ* hybridization results are consistent with the qRT-PCR results shown in Fig. 7B. All larvae were at 84 HPF (hours postfertilization).

Fig. S1.

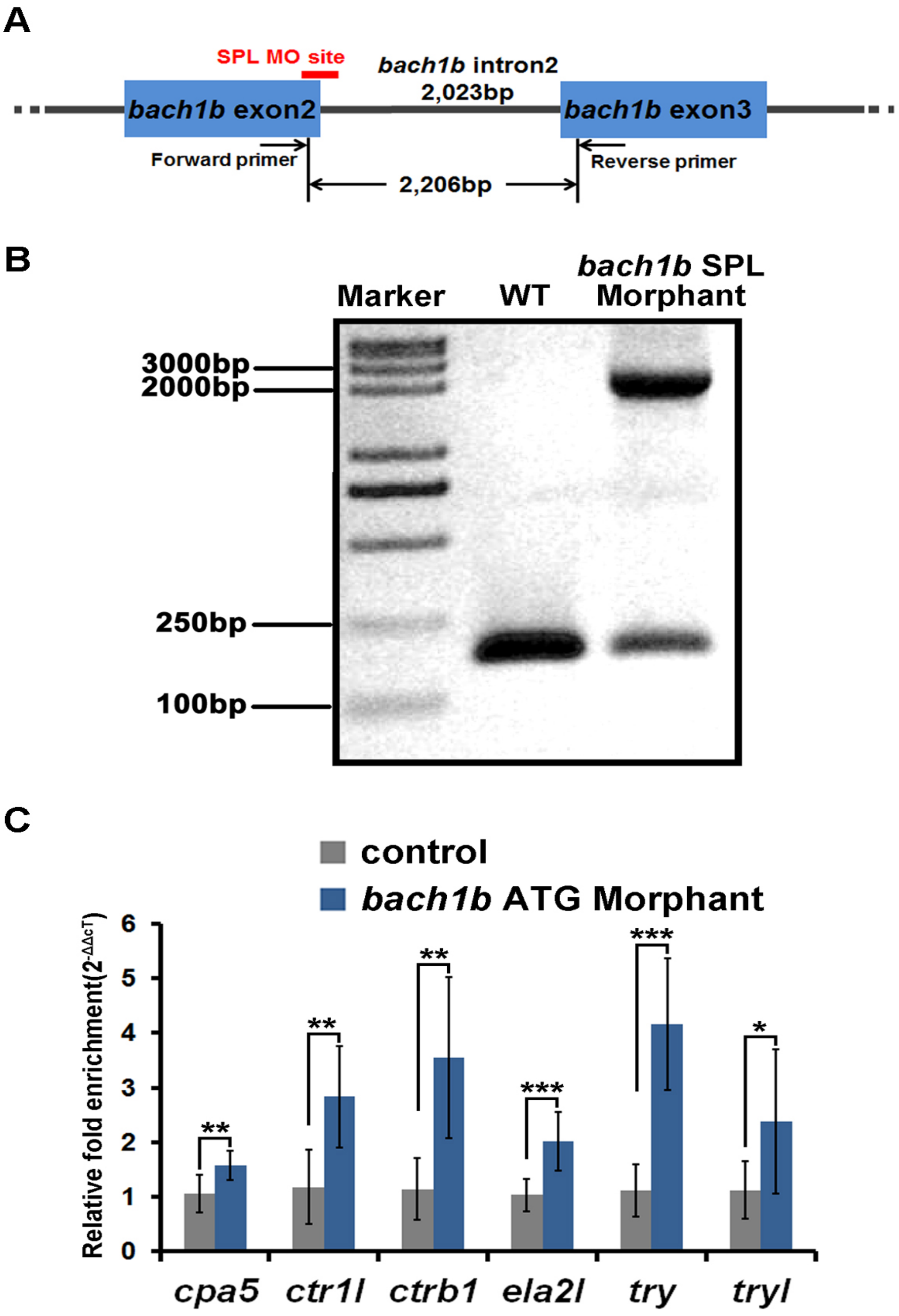
A
SPL MO site
bach1b intron2
2,023bp
bach1b exon2
bach1b exon3
Forward primer
Reverse primer
2,206bp
B
Marker
WT
bach1b SPL Morphant
3000bp
2000bp
250bp
100bp
C
control
bach1b ATG Morphant
Relative fold enrichment($2^{-\Delta\Delta cT}$)
0
1
2
3
4
5
6
**
**
**
***
***
*
cpa5
ctr1l
ctrb1
ela2l
try
tryl

Fig. S2.

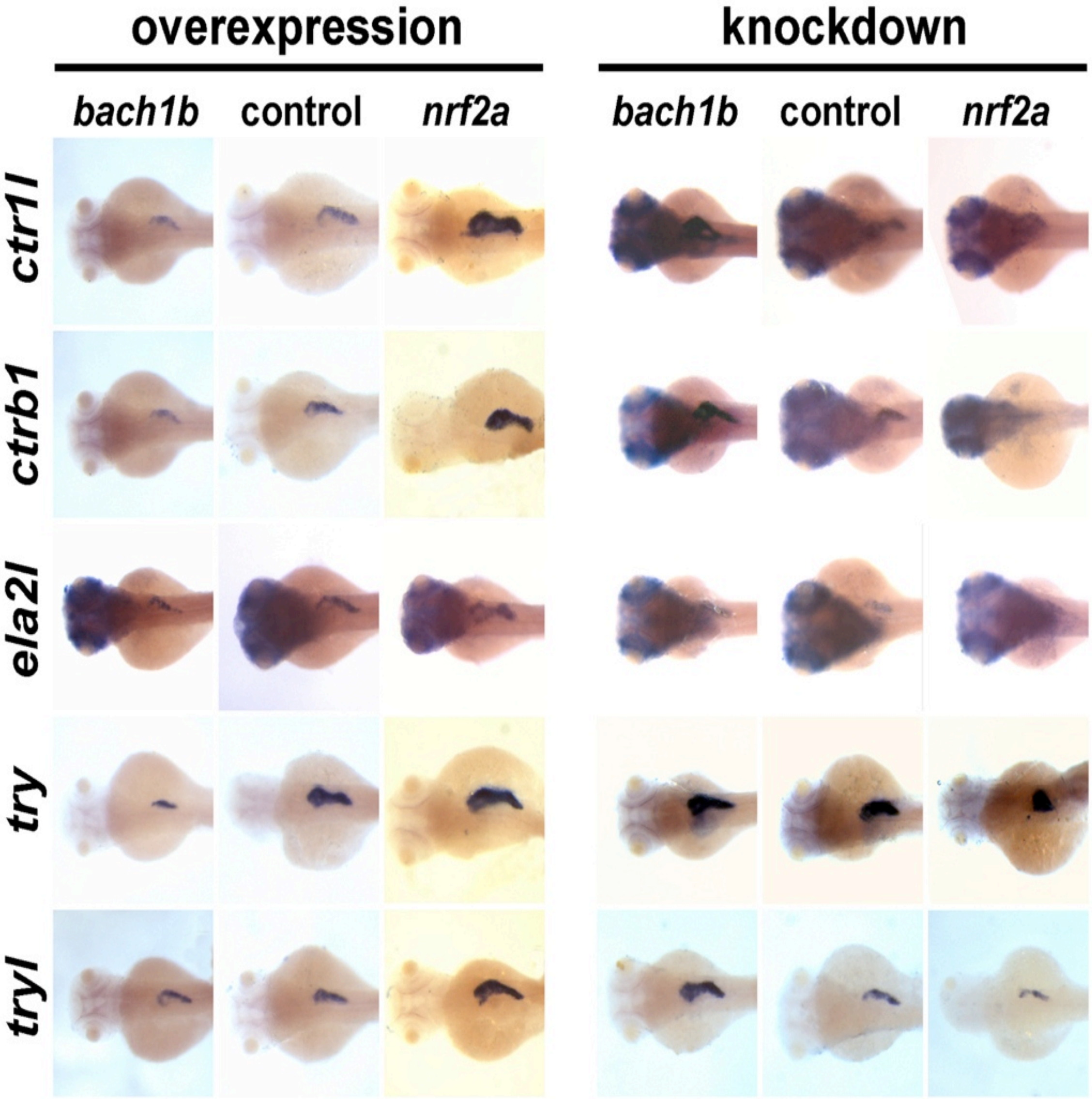
overexpression
knockdown
bach1b
control
nrf2a
bach1b
control
nrf2a
ctr1l
ctrb1
ela2l
try
tryl

Fig. S3.

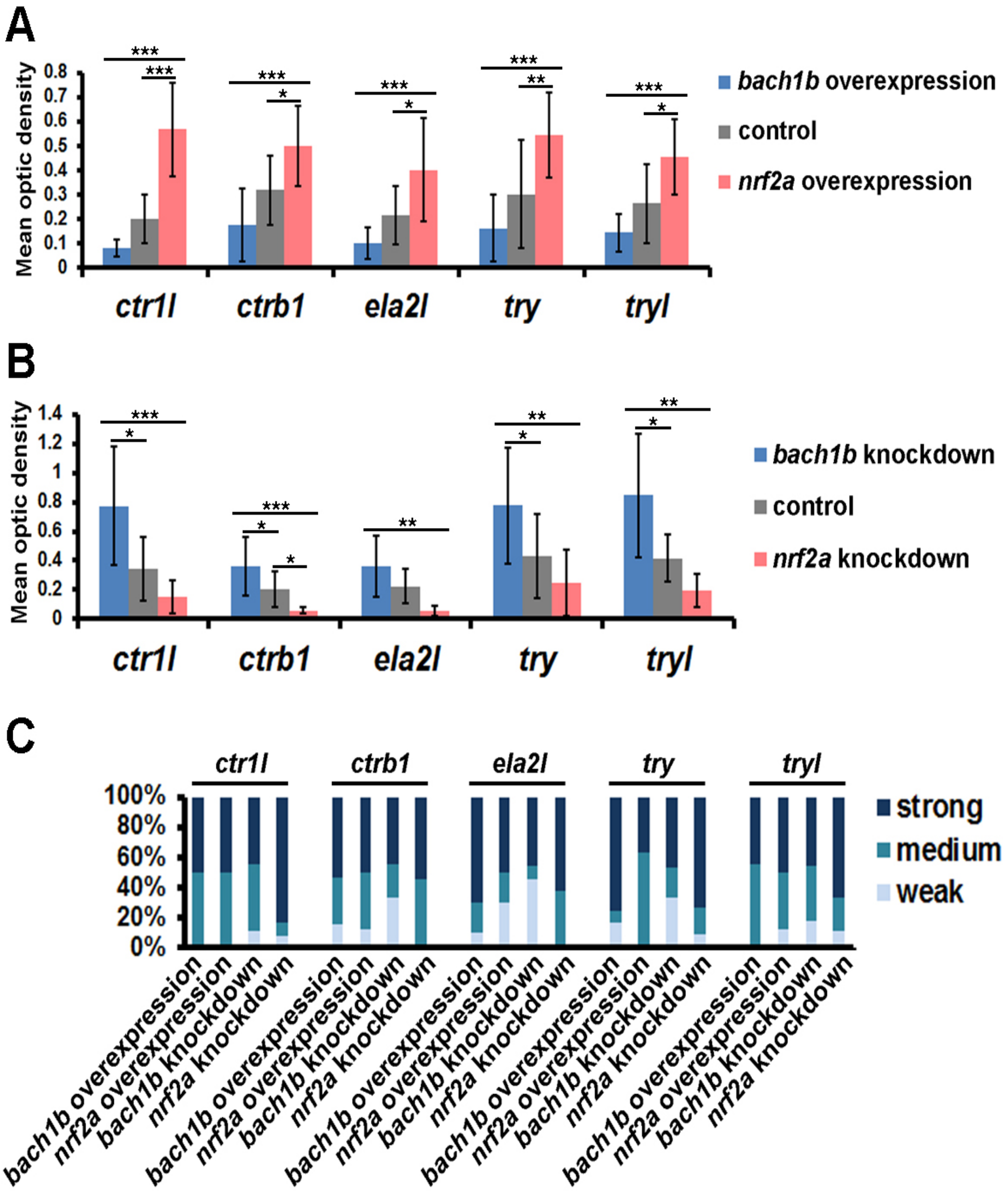
A
Mean optic density
0.8
0.7
0.6
0.5
0.4
0.3
0.2
0.1
0
***
**
*
ctr1l
ctrb1
ela2l
try
tryl
bach1b overexpression
control
nrf2a overexpression
B
Mean optic density
1.4
1.2
1
0.8
0.6
0.4
0.2
0
bach1b knockdown
control
nrf2a knockdown
C
100%
80%
60%
40%
20%
0%
strong
medium
weak
bach1b overexpression
nrf2a overexpression
bach1b knockdown
nrf2a knockdown

Fig. S4.

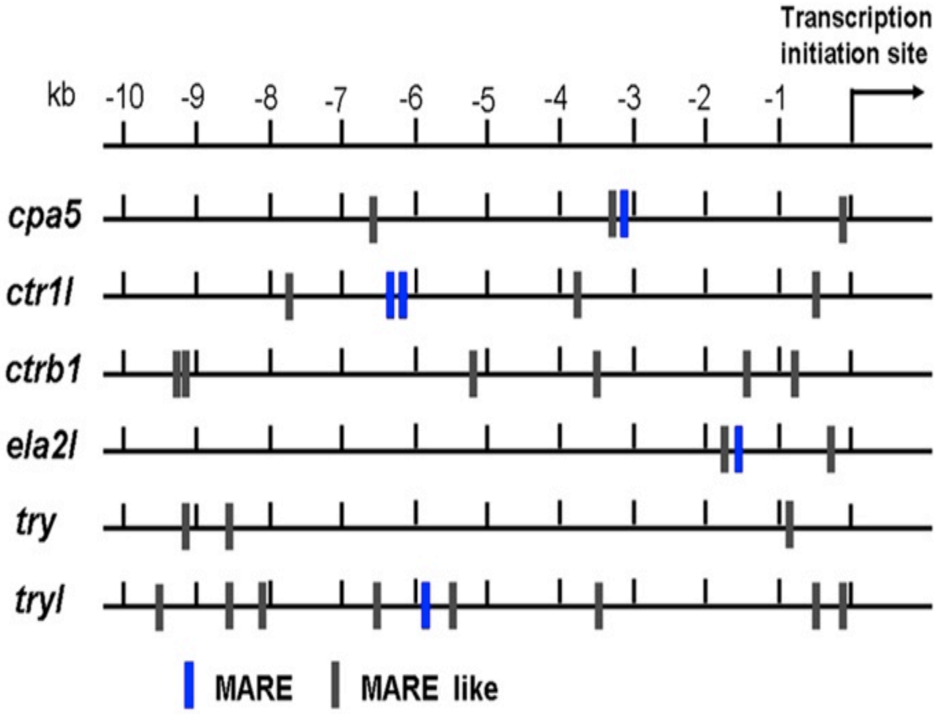
Transcription initiation site
kb
-10
-9
-8
-7
-6
-5
-4
-3
-2
-1
cpa5
ctr1l
ctrb1
ela2l
try
tryl
MARE
MARE like

Fig. S5.

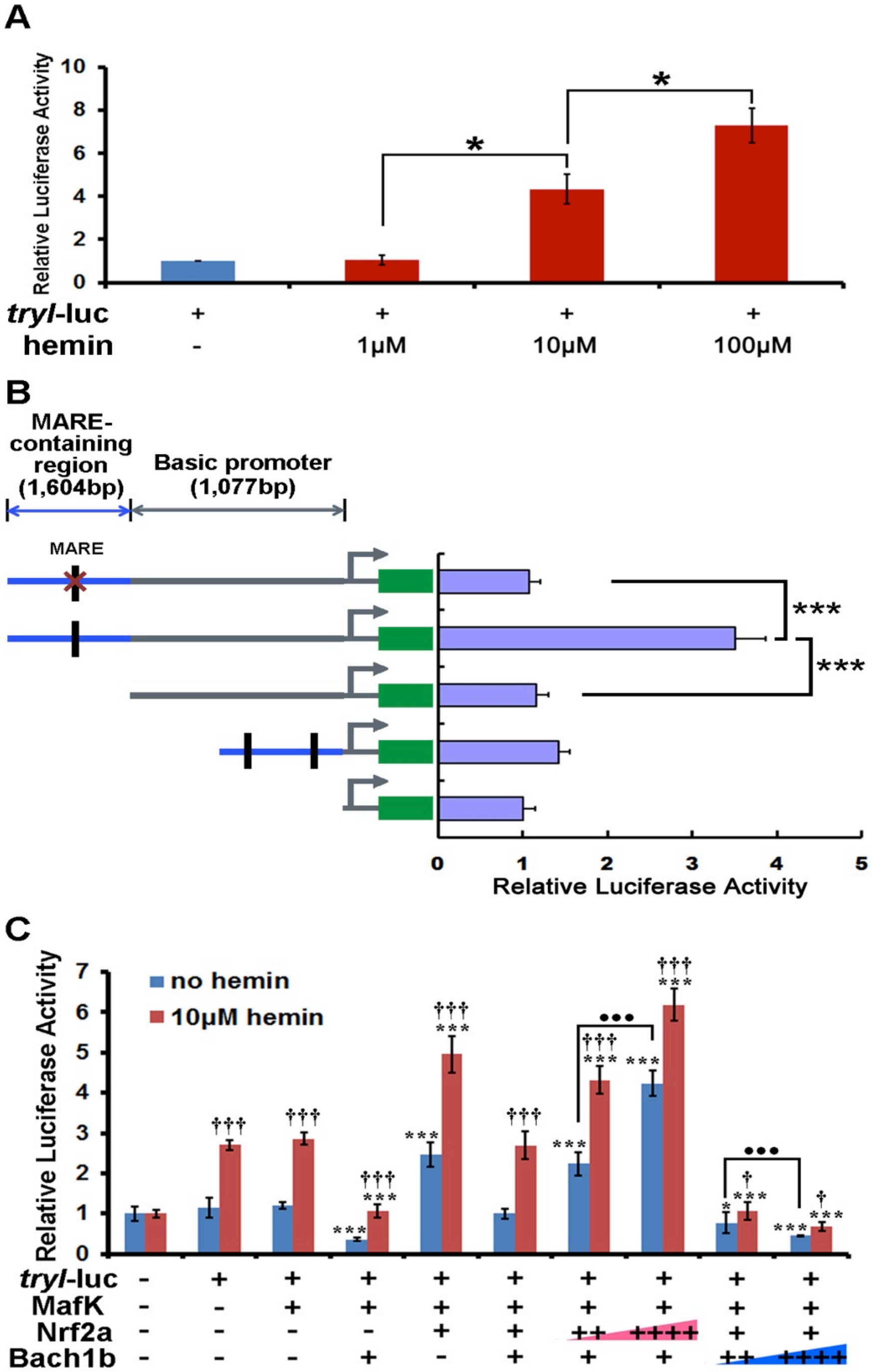
A
Relative Luciferase Activity
0
2
4
6
8
10
*
*
tryl-luc
+
+
+
+
hemin
-
1μM
10μM
100μM
B
MARE-containing region (1,604bp)
Basic promoter (1,077bp)
MARE
***
***
0
1
2
3
4
5
Relative Luciferase Activity
C
no hemin
10μM hemin
Relative Luciferase Activity
tryl-luc
MafK
Nrf2a
Bach1b

Fig. S6.

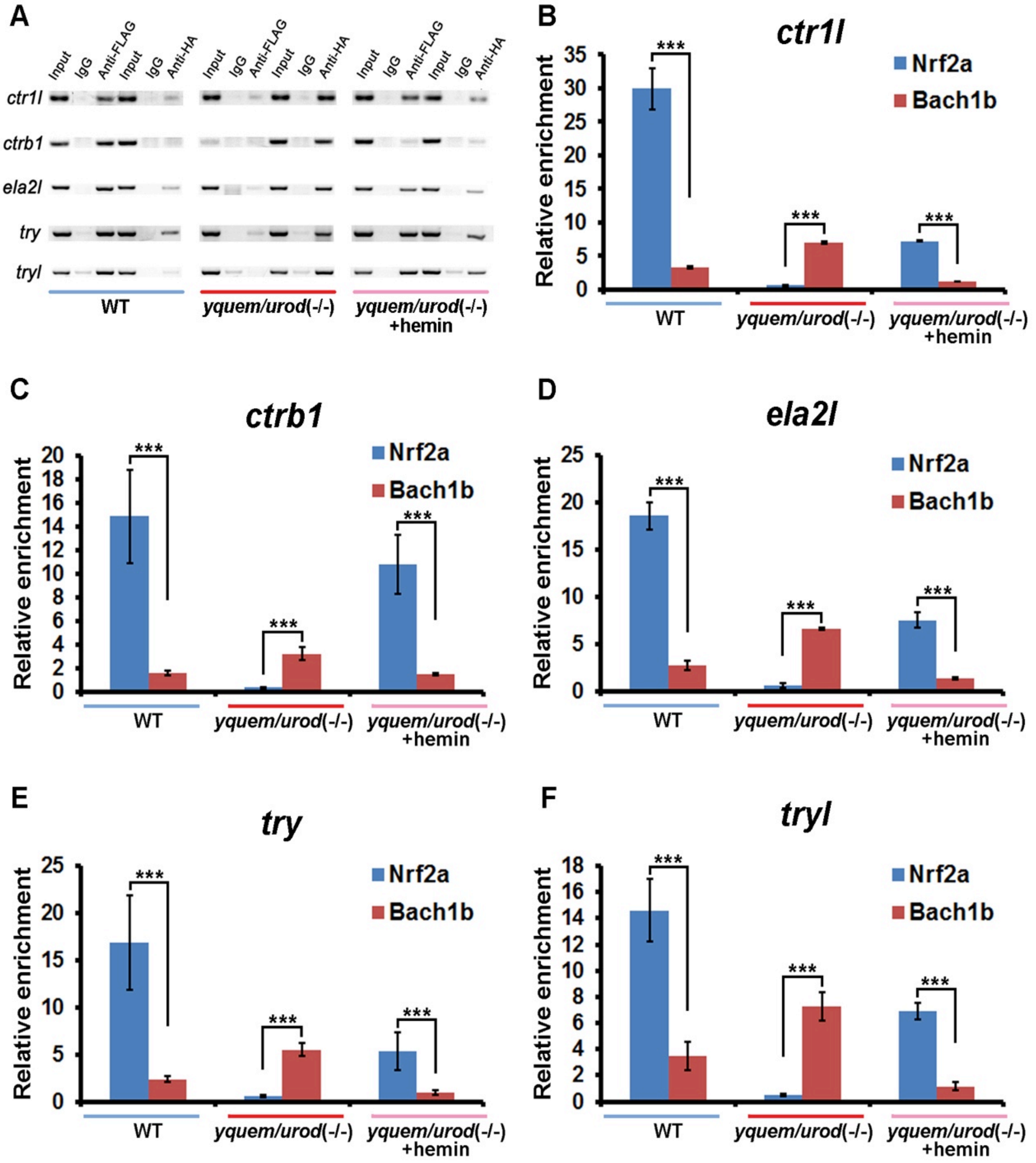
A
Input
IgG
Anti-FLAG
Input
IgG
Anti-HA
ctr1l
ctrb1
ela2l
try
tryl
WT
yquem/urod(-/-)
yquem/urod(-/-) +hemin
B
ctr1l
Nrf2a
Bach1b
Relative enrichment
***
C
ctrb1
D
ela2l
E
try
F
tryl

Fig. S.7.

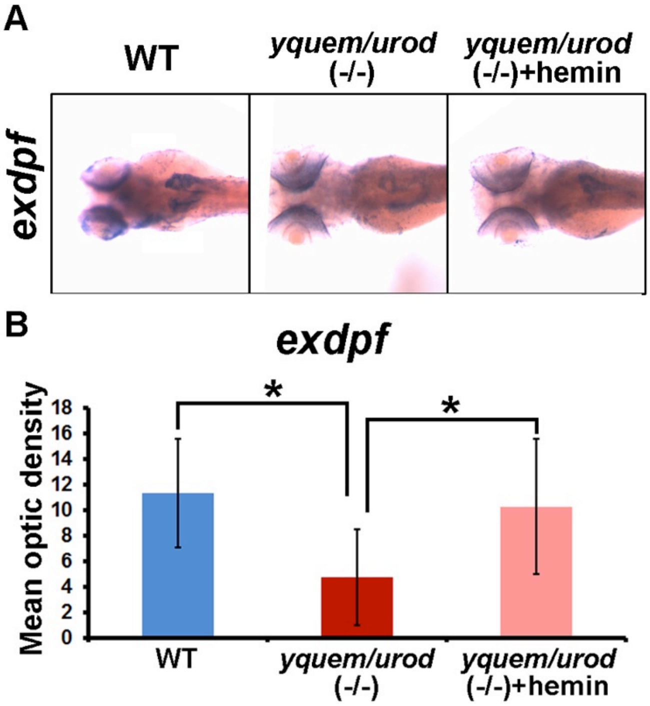
A
WT
yquem/urod (-/-)
yquem/urod (-/-)+hemin
exdpf
B
exdpf
Mean optic density
18
16
14
12
10
8
6
4
2
0
*
*
WT
yquem/urod (-/-)
yquem/urod (-/-)+hemin
